# PERK-dependent reciprocal crosstalk between ER and non-centrosomal microtubules coordinates ER architecture and cell shape

**DOI:** 10.1016/j.celrep.2025.115590

**Published:** 2025-04-22

**Authors:** Miguel Sánchez-Álvarez, Fidel Nicolás Lolo, Heba Sailem, Giulio Fulgoni, Patricia Pascual-Vargas, Lucía Agüera, Mauro Catalá-Montoro, Mar Arias-García, Juan Antonio López, Jesús Vázquez, Miguel Ángel del Pozo, Chris Bakal

**Affiliations:** 1 Dynamical Cell Systems Team, Division of Cancer Biology, The Institute of Cancer Research-Chester Beatty Laboratories, 237 Fulham Road, London SW3 6JB, UK; 2 Cell Compartmentalization, Homeostasis and Inflammation Team, Department of Metabolic and Inflammatory Diseases, Instituto de Investigaciones Biomédicas “Sols-Morreale”, CSIC-UAM, CP 28029 Madrid, Spain; 3 Mechanoadaptation and Caveolae Biology Laboratory, Area of Cell and Developmental Biology, Centro Nacional de Investigaciones Cardiovasculares (CNIC), c/Melchor Fernandez Almagro, 3, CP 28029 Madrid, Spain; 4 Proteomics Unit, Centro Nacional de Investigaciones Cardiovasculares (CNIC), c/Melchor Fernandez Almagro, 3, CP 28029 Madrid, Spain; 5 Cardiovascular Proteomics Lab, Centro Nacional de Investigaciones Cardiovasculares (CNIC), c/Melchor Fernandez Almagro, 3, CP 28029 Madrid, Spain; 6 CIBER de Enfermedades Cardiovasculares (CIBERCV), Madrid, Spain

**Keywords:** endoplasmic reticulum, integrated stress response, EIF2AK3/PERK, non-centrosomal microtubules, cell polarity

## Abstract

The architecture of the endoplasmic reticulum (ER) is a key determinant of its function. Its dynamics are linked to those of the cytoskeleton, but our understanding of how this coordination occurs and what its functional relevance is, limited. Here, we report that the unfolded protein response (UPR^ER^) transducer EIF2AK3/PERK (eukaryotic translation initiation factor 2-alpha kinase 3/protein kinase R-like endoplasmic reticulum kinase) is essential for acute-stress-induced peripheral redistribution and remodeling of the ER through eukaryotic initiation factor 2 alpha (eIF2α) phosphorylation and translation initiation shutdown. PERK-mediated eIF2α phosphorylation can be bypassed by blocking polysome assembly, depleting microtubule (MT)-anchoring ER proteins such as p180/RRBP1 (ribosome-binding protein 1), or disrupting the MT cytoskeleton. Specific disruption of non-centrosomal MTs, but not centrosome depletion, rescues ER redistribution in PERK-deficient cells. Conversely, PERK deficiency stabilizes non-centrosomal MTs against proteasomal degradation, promoting polarized protrusiveness in epithelial cells and neuroblasts. Thus, PERK coordinates ER architecture and homeostasis with cell morphogenesis by coupling ER remodeling and non-centrosomal MT stability and dynamics.

## Highlights

• PERK/EIF2AK3 is identified as a regulator of ER architecture remodeling
• Translation initiation suppression is required for ER remodeling during stress
• Regulation of ER anchoring to non-centrosomal MTs is required for ER remodeling
• ER-MT anchoring feeds back onto cell morphology through non-centrosomal MTs

## Graphical abstract

**Figure:**
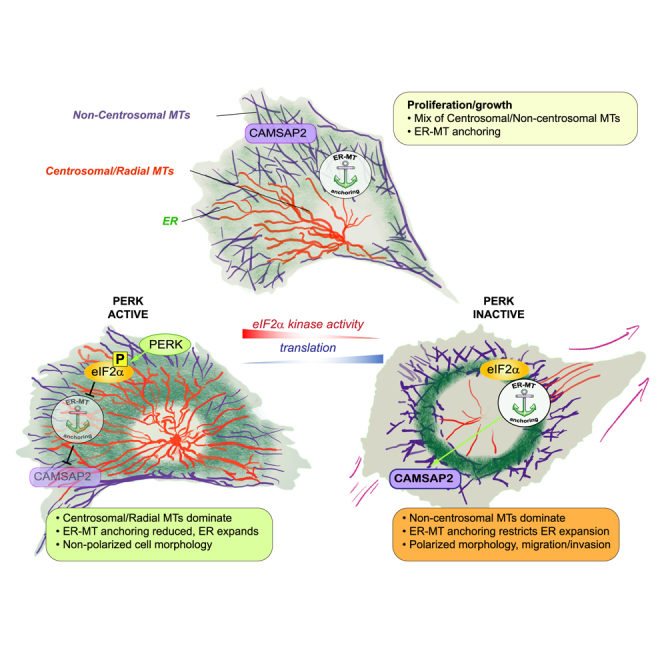

## Teaser

Sanchez-Alvarez et al. report that ER anchoring to non-centrosomal microtubules is regulated by PERK translation suppression activity to both enable ER architecture remodeling during stress and modulate back microtubule dynamics by determining their stability. This mechanism thus couples cell polarization and protrusiveness with ER homeostasis.

## Introduction

The eukaryotic endoplasmic reticulum (ER) serves several essential functions, including calcium and redox homeostasis, complex lipid metabolism, and the maturation and assisted folding of ∼30% of the proteome.^1^ ER membrane subdomains can adopt discrete shapes (including ER “tubules” [peripheral, reticular tubes of ER with rather low densities of associated ribosomes] and ER “sheets” [flat enlargements or “cisternae” of peripheral ER usually rich in bound polysomes])^2^; this model may oversimplify a more complex variety of ER architectures.^3^ The local concentration and activity of “ER shapers” such as reticulons, atlastins, and other auxiliary resident proteins^4^ determine the shape of the ER. Physical expansion is an integral component of the ER adaption to functional imbalances (“ER stress”)^5^ through the unfolded protein response of the ER (UPR^ER^), a surveillance mechanism that continuously gauges the ER luminal environment and membrane integrity and engages adaptive programs as required.^6^^,^^7^^,^^8^

The UPR^ER^ of higher eukaryotes comprises three main branches, each of them driven by a specific, ER-resident transducer. Inositol-requiring enzyme 1 (IRE1; also known as endoplasmic reticulum to nucleus signaling 1 [ERN1]) orchestrates a complex adaptive transcriptional response through the unconventional splicing of *XBP1* (X-box binding protein 1) mRNA, eliciting its translation as a potent transcriptional transactivator of ER chaperones and lipid anabolism.^8^^,^^9^ Upon activated intramembrane cleavage, activation transcription factor 6 (ATF6) also promotes adaptive transcriptional programs, driving redox regulation, ER chaperone expression, and lipid metabolism enzymes.^8^^,^^10^ Both UPR branches are involved in ER membrane *de novo* synthesis and ER physical expansion.^11^^,^^12^^,^^13^ The third branch is operated by the eukaryotic translation initiation factor 2-alpha kinase 3/protein kinase R (PKR)-like endoplasmic reticulum kinase (EIF2AK3/PERK), one of the four known stress-associated kinases in metazoans capable of phosphorylating the essential translation regulator eukaryotic initiation factor 2 alpha (eIF2α). eIF2α is a GTPase subunit of the translation initiation ternary complex^8^; phosphorylation on its conserved Ser51 residue locks eIF2α in its GDP-bound, inactive state and leads to sequestration of the ternary complex from further assembling a processive ribosome. eIF2α phosphorylation is a node onto which several stress responses (including UPR^ER^ through PERK) converge to curb protein synthesis. Limiting client protein entry into the stressed ER through translation downregulation is commonly proposed as the main role of PERK.^8^^,^^14^ However, PERK-dependent protein translation attenuation is integrated with other functional outputs, such as regulation of calcium trafficking and apoptosis.^15^^,^^16^^,^^17^

The ER communicates with other organelles, plasma membrane subdomains, and the cytoskeleton.^18^ The metazoan ER is tightly coupled to the microtubule (MT) cytoskeleton.^19^^,^^20^^,^^21^^,^^22^ Several ER membrane proteins, such as ribosome-binding protein 1 (RRBP1/p180), cytoskeleton-linking membrane protein 63 (Climp63/CKAP4), and receptor expression-enhancing proteins 1–4 (REEPs 1–4) or reticulons, can engage in physical contact with MTs.^23^^,^^24^^,^^25^^,^^26^^,^^27^^,^^28^ Extension, fusion, and reticulation of ER tubules are guided by dynamic MT bundles.^18^ ER sheets also establish anchoring interactions with MTs, preferentially through p180/RRBP1 and Climp63, which in turn also establish interactions with polysome/translocon complexes enriched in these areas of “rough” ER.^29^^,^^30^ These interactions modulate both ER morphology and MT organization; for example, p180/RRBP1 overexpression can induce hyperstabilization of MT bundles and promote the formation of tight, collapsed structures.^24^

We developed image-based RNAi screening procedures to explore the genetic regulation of ER expansion and redistribution upon pharmacologically challenging ER homeostasis. We found that PERK is essential for ER redistribution to the cell periphery during ER expansion upon induction of ER stress. This activity is dependent on translation initiation shutdown through eIF2α phosphorylation and can be bypassed through pharmacological blockade. Combinatorial small interfering RNA (siRNA) screening revealed that the depletion of proteins linking MTs with the ER, such as REEP4, p180/RRBP1, and Climp63/CKAP4, specifically rescued the ER phenotype associated with impaired PERK signaling. Importantly, while centrosome depletion did not have an observable impact on ER architecture dynamics, the depletion of non-centrosomal MTs through the knockdown of calmodulin-regulated spectrin-associated protein family member 2 (CAMSAP2) promoted the peripheral expansion of ER non-tubular structures and fully rescued ER redistribution in PERK-depleted cells. Conversely, abrogation of PERK activity stabilized non-centrosomal MTs and led to phenotypes of increased polarity and low numbers of large protrusions in epithelial cells. Mechanistically, the physical anchoring of the ER to non-centrosomal MTs promotes the stabilization of the latter, as well as of CAMSAP2 itself. During ER stress, PERK inhibits translation initiation, which attenuates the anchoring of the ER to non-centrosomal MTs through specific ER-MT linkers such as RRBP1/p180, enabling ER expansion and modulating MT cytoskeleton arrangement. Our observations highlight the existence of additional key roles for PERK on cell homeostasis beyond the curbing of ER client protein synthesis.

## Results

### PERK regulates ER redistribution during acute ER stress in epithelial cells

**Figure 1:**
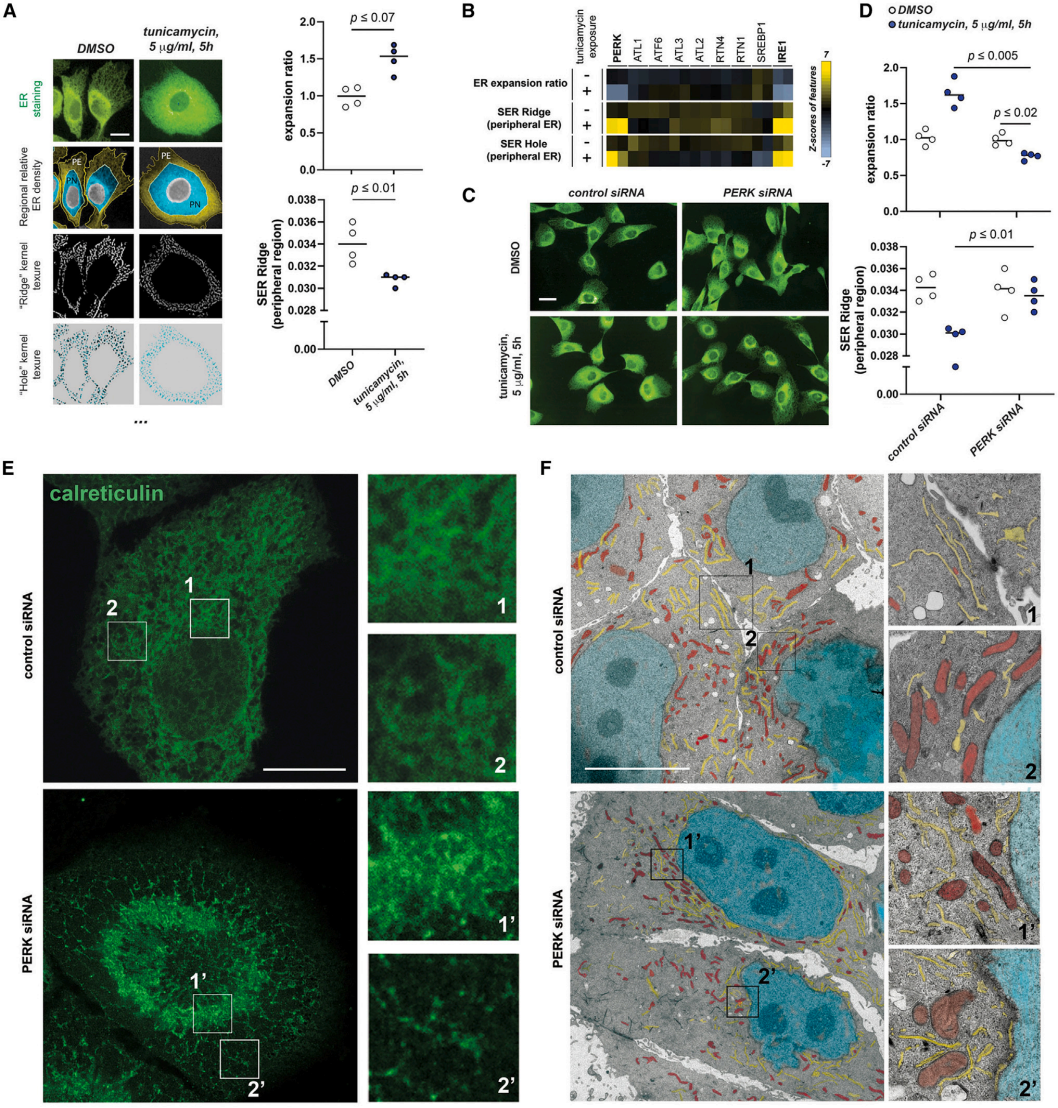
An automated image-based assay identifies EIF2AK3/PERK as required for ER remodeling during acute ER stress (A) Automated image analysis features informative of ER architecture remodeling in MCF10A cells treated as indicated from unprocessed immunofluorescence images of total ER (anti-calreticulin immunostaining). Graphs (right) are derived from four independent replicates. PN (perinuclear region) and PE (peripheral region) areas for the calculation of regional relative ER density (hereafter the “expansion ratio”) are established with constant relative distances between the nuclear boundary and the cell edge. The two bottom images and the bottom graph exemplify image texture features (calculated as fitting ridge or hole kernels of 1 px width). Scale bar represents 10 μm. Related supplemental information on the high-content data and procedure is contained in Figures S1, Table S1, and Data S1. (B–D) Automated image-based exploration of genetic regulators of ER-stress-driven ER remodeling using pools of 4 siRNA duplexes to interrogate each chosen gene. (B) Heatmaps (*Z* scores) of selected features, informative for ER remodeling, across conditions, as compared to control cells. (C) Representative images for indicated conditions. (D) Graphs derived from well-normalized values for indicated features across conditions (four independent replicates). Scale bar represents 15 μm. (E and F) STED microscopy (calreticulin immunostaining) (E) and electron microscopy (F) of MCF10A cells across indicated conditions. Magnified cropped images show details of the indicated rectangular example regions of interest (ROIs) at perinuclear or peripheral cell regions. Pseudocoloring in (F): cyan, nuclei; red, mitochondria; yellow, ER. *p* values are indicated across experiments. Scale bars represent 10 μm in (E) and 5 μm in (F). Statistical significance values from Student’s t tests are indicated as ^∗^*p* < 0.05, ^∗∗^*p* < 0.01, and ^∗∗∗^*p* < 0.005. n.s. *p* > 0.05. Bar graph items show mean values (bar graphs) and standard deviation (error bars); dot plots represent individual replica values, with their average indicated with a horizontal bar. Heatmap represents *Z* scores as related to control average and standard deviation for each feature.

We developed an image analysis pipeline for automated high-content ER morphology analysis in single cells captured by spinning disk confocal microscopy (Figure 1A).^31^^,^^32^ An array of ER-related features (relative distribution ratio between peripheral and perinuclear regions and image texture features) are extracted from single cells, together with other morphological features of the whole cell (Figures 1A and S1A; see also Data S1). We first compared wild-type cells with cells exposed to the N-glycosylation inhibitor tunicamycin, which provokes acute ER stress and a prominent redistribution and expansion of the ER in epithelial cells, together with changes in image texture (Figure 1A). The ratio of ER signal density on the cell periphery to that at the perinuclear region was informative of adaption to ER stress (Figure 1A, right).^28^^,^^31^ In our analysis, the relative extension and positioning of these two distinct cell regions are established with a fixed relationship to cell area and the distance between the plasma membrane and nucleus periphery, rendering this parameter robust to variations in cell shape and/or size (see STAR Methods); robust automated focal plane positioning is yet another advantage of our approach regarding the comparison of ER architecture across conditions. Image texture as related to specific kernels (“ridges” or “holes”) further captured in our cell system, at this image resolution, significant changes in peripheral ER architecture upon tunicamycin challenge (Figure 1A, bottom and plot). We further tested this system by interrogating, both in untreated and tunicamycin-treated cells, a small collection of siRNAs, targeting well-established direct regulators of ER morphogenesis and homeostasis (see Table S1). Hierarchical clustering across image features correctly grouped both siRNA species tested for each gene (Figure S1A). The knockdown of IRE1α, a key regulator of ER membrane expansion,^5^^,^^33^ led to reduced peripheral ER distribution across conditions (Figures 1B and S1B). We propose that our methodology captures physiologically relevant changes in ER morphology in single cells during ER stress.

Surprisingly, we found that depletion of the PERK (PERK/EIF2AK3), a well-established essential factor for ER homeostasis,^14^ led to a marked impairment for ER subcellular redistribution upon exposure to tunicamycin (Figures 1B–1D and S1A). Four different siRNA sequences were chosen for secondary validation; their effectiveness was assessed by RT-qPCR (Figure S1C). Transfection of all four deconvoluted siRNAs recapitulated the phenotype of impaired peripheral ER redistribution upon tunicamycin challenge (Figure S1D). Stimulated emission depletion (STED) microscopy and electron microscopy confirmed that the ER of cells depleted of PERK is not rearranged to increase peripheral sheets in response to ER stress but rather collapses toward the perinuclear region (Figures 1E, 1F, and S1E). Furthermore, the phenotype of impaired ER subcellular redistribution upon acute exposure to tunicamycin could also be followed through live imaging of a stable MCF10A-derived cell line expressing EGFP-tagged Sec61β (Figure S1F). PERK knockdown in other cell lines of different origins, such as a transformed MCF10A AT clone or two other tumor epithelial cell lines (HeLa and MDA-MB231), resulted in analogous phenotypes (Figure S1G).

**Figure 2:**
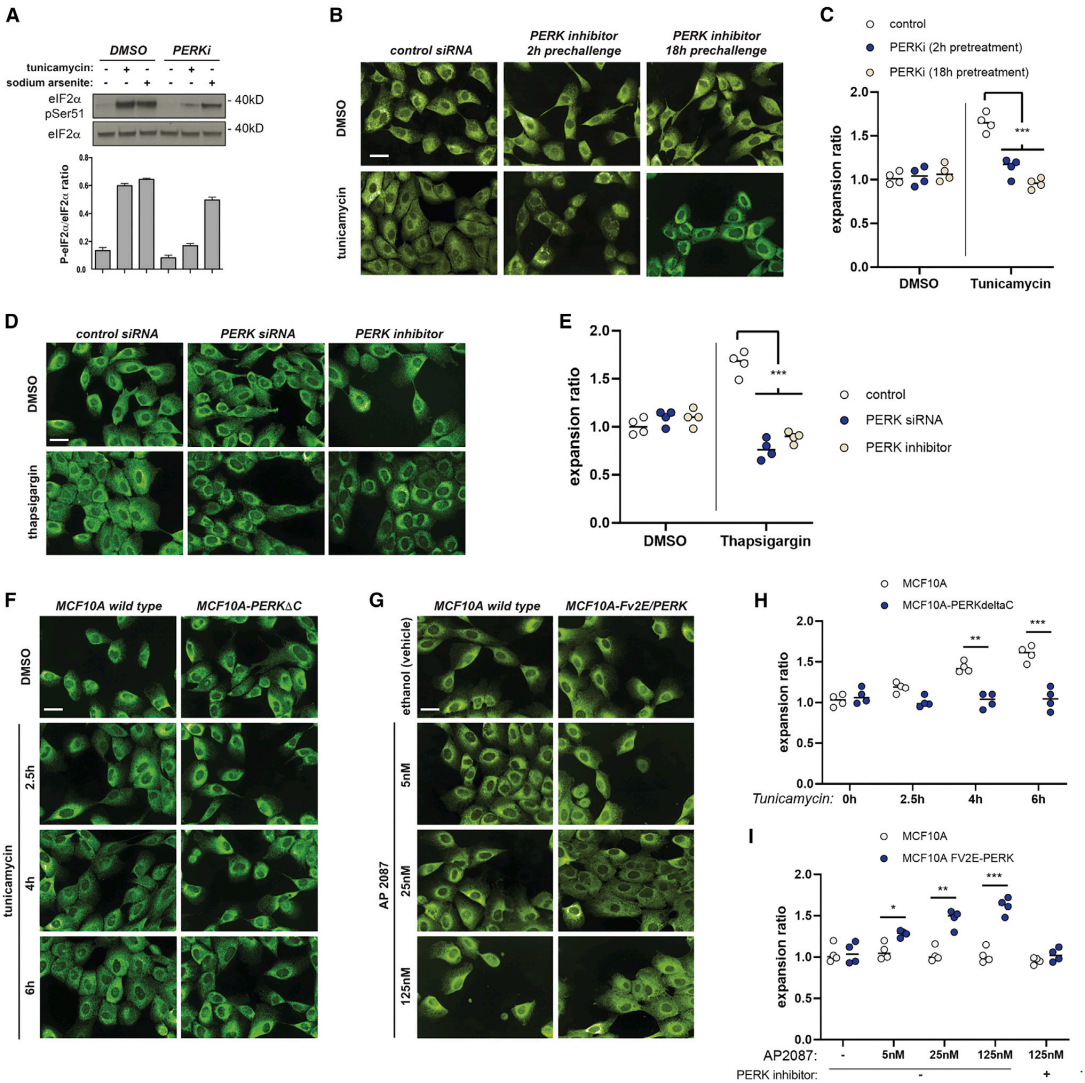
PERK is an essential effector of acute ER-stress-driven ER remodeling (A–C) Allosteric inhibition of PERK kinase activity recapitulates the phenotype observed upon siRNA-mediated PERK depletion. (A) Assessment of the activity and specificity of the inhibitory compound (PERKi; GSK2606414, see STAR Methods) by western blot analysis of whole-cell lysates. (B and C) Representative images (calreticulin immunostaining) (B) and data derived from four biological replicates (∼2,000 cells per well) (C) across indicated treatment conditions. (D and E) Representative anti-calreticulin immunofluorescence staining across indicated conditions (D) and data derived from four biological replicates (∼2,000 cells per well) across indicated treatments (E). (F and H) Stable expression of a dominant-negative PERK-ΔC truncated mutant blunts ER peripheral remodeling. Data are derived from four biological replicates (∼2,000 cells per well) across indicated treatments. (G and I) Exposure of cells expressing a PERK construct fused with an homodimerizing domain (PERK-Fv2E) to the AP20187 homodimerizer enables PERK kinase-dependent ER remodeling to bypass ER stress induction. Data are derived from four biological replicates (∼2000 cells per well) across indicated treatments. All scale bars across micrographs represent 15 μm. Statistical significance values from Student’s t tests are indicated as ^∗^*p* < 0.05, ^∗∗^*p* < 0.01, and ^∗∗∗^*p* < 0.005. n.s. *p* > 0.05. Bar graph items show mean values (bar graphs) and standard deviation (error bars); dot plots represent individual replica values, with their average indicated with a horizontal bar.

We exposed cells to the allosteric PERK kinase inhibitor GSK2606414 (Figure 2A) for different amounts of time prior to tunicamycin challenge. These pretreatments recapitulated the ER collapse associated with PERK siRNA-mediated depletion during ER stress (Figures 2B and 2C). Disruption of PERK kinase activity in unchallenged cells also led to moderate phenotypic alterations regarding cell elongation and peripheral ER architecture, similar to those observed for PERK siRNA-mediated knockdown. PERK siRNA transfection or exposure to the PERK kinase inhibitor was also associated with ER collapse when cells were challenged with a different source of ER stress, such as the sarco/ER Ca2+-ATPase inhibitor thapsigargin (Figures 2D and 2E).

We further tested two established cell models, derived from the MCF10A epithelial line, that reprogram PERK regulation and output. First, we looked into the phenotype of the PERK-ΔC cell line—which expresses a C-terminal truncated, dominant-negative mutant^34^^,^^35^—across different conditions. The PERK-ΔC-expressing cell line exhibited partially impaired redistribution of its ER upon tunicamycin exposure (Figures 2F and 2G). We studied the behavior of another MCF10A-derived cell line, Fv2E-PERK, which expresses a synthetic PERK construct by which it is possible to uncouple PERK activation from canonical ER stress^34^^,^^35^ (Figure S2A). Exposure to the B/B homodimerizer AP20187 provoked apparent ER peripheral expansion in Fv2E-PERK cells in the absence of ER stressors but not in wild-type MCF10A cells (Figures 2H and 2I). These observations support that the phenotypes we observe are specifically derived from altered PERK kinase activity.

### PERK/EIF2AK3 kinase role in peripheral ER architecture remodeling does not target *de novo* ER membrane synthesis and depends on eIF2α phosphorylation

In certain cell models, PERK is potentially required for the activation of the lipid anabolism regulator sterol regulatory element binding protein 1 (SREBP1).^36^ We hypothesized that the observed deficiency in ER expansion in PERK-deficient cells could be due to impaired lipid anabolism and/or *de novo* membrane synthesis. However, siRNA-mediated depletion of PERK did not significantly impact the activation of canonical ER lipid anabolism regulators such as SREBP1 or XBP1^11^^,^^12^^,^^13^^,^^36^ (Figure S2B). Cells depleted for these regulators exhibited ER phenotypes different from those displayed by cells knocked down for PERK (see Figure S1B). The increase in total ER membrane content upon ER stress induction in PERK-depleted cells is comparable to that of wild-type cells (Figure S2C). Supplementation of a traceable modified choline precursor revealed that PERK-deficient cells are not impaired for phosphatidylcholine *de novo* incorporation (Figure S2D; see STAR Methods), a key event sustaining ER-stress-induced ER membrane expansion.^12^ These observations support a potential novel role for PERK in the control of ER architecture remodeling and subcellular redistribution during ER stress, distinct from *de novo* ER membrane synthesis.

ATF4 is a key UPR transcriptional transducer whose translation is dependent on the ribosomal frameshift elicited by PERK-dependent eIF2α phosphorylation.^16^ PERK depletion in our cell system blunted ER-stress-driven ATF4 translation (Figure S2E), but siRNA-mediated depletion of ATF4 did not recapitulate the phenotypic characteristics associated with the abrogation of PERK activity and, in fact, led to moderate but significantly opposite effects regarding ER remodeling, cell spreading, and overall morphology across the tested conditions (Figures S2E and S2F). These observations support the notion that the role of PERK in the phenotypes we observe is not dependent on downstream gene expression programs driven by ATF4.

**Figure 3:**
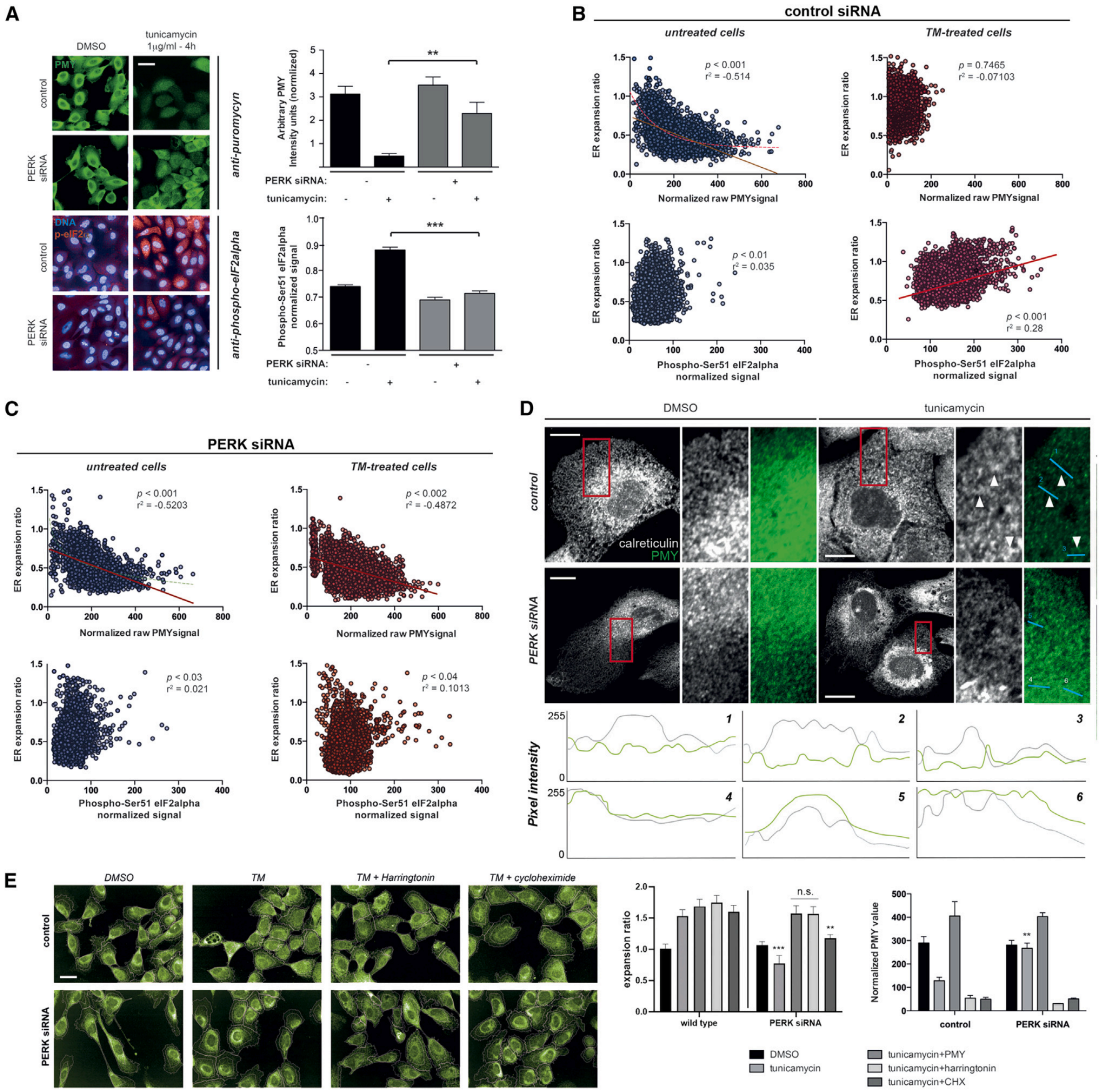
PERK-dependent translation initiation inhibition correlates with peripheral ER remodeling (A) Puromycylation assays reveal a correlation between PERK-dependent protein translation shutdown and eIF2α phosphorylation upon exposure to acute ER stress in MCF10A cells. Data are derived from eight biological replicates (∼2,000 cells per well) across indicated treatments for each label. Scale bar represents 15 μm. (B and C) Plotting of values recorded for single MCF10A cells from cultures either mock transfected (B) or PERK depleted (C). Note the correlation and statistical significance of this correlation across treatments between labels and how this relationship is no longer sensitive to ER stress induction in PERK-depleted cells. At least 800 cells per condition were recorded. (D) Single confocal planes of MCF10A cells stained for ER (calreticulin; grayscale) and active translation (PMY; green pseudocoloring) across indicated siRNA and compound treatments. Peripheral sheet-like patches in mock-transfected cells exposed to ER stress depleted of the PMY label are indicated by arrowheads. Intensity profiles across the indicated lines are shown below. All scale bars represent 5 μm. (E) PMY stands for both the puromycylation label and sustained inhibitory treatment. CHX, cycloheximide. Treatments (harringtonin, 500 ng/mL; PMY, 1 μg/mL; and CHX, 20 μg/mL) were applied for the last 60 min of all experiments. Data are derived from four biological replicates (∼2,000 cells per well) across indicated treatments. Scale bar represents 15 μm. Statistical significance values from Student’s t tests are indicated as ^∗^*p* < 0.05, ^∗∗^*p* < 0.01, and ^∗∗∗^*p* < 0.005. n.s. *p* > 0.05. Bar graph items show mean values (bar graphs) and standard deviation (error bars); dot plots represent individual cell values with lines indicating linear regression analyses.

We implemented a method to quantify translation activity and ER subcellular distribution and architecture on a single-cell basis. Polysomes engaged in active translation are specifically immunolabeled and visualized after a brief pulse of puromycin (puromycylation, hereafter called “PMY”; Figures S3A and S3B).^37^ Acute induction of ER stress leads to a substantial decrease in the PMY signal, which correlates with the upregulation of the phosphor-eIF2α signal, in a PERK-dependent manner (Figure 3A). We plotted ER distribution against the PMY signal across different conditions for single wild-type MCF10A cells (Figure 3B). Importantly, translation activity (as judged by PMY intensity) and degree of relative peripheral ER density exhibited an *inverse* correlation on a single-cell basis. Moreover, the phosphor-eIF2α signal (inhibition of translation) followed an opposite (positive) correlation pattern with ER expansion on a single-cell basis (Figure 3B, bottom). Thus, acute induction of ER stress robustly increased ER expansion but suppressed global translation (Figure 3B, top).

The knockdown of PERK altered these responses (Figure 3C). Peripheral expanded ER structures in wild-type cells subject to ER stress showed reduced overlap with the PMY signal (Figure 3D, intensity line plots 1–3), supporting that translational shutdown is a required step for ER adaptive remodeling. In contrast, PERK-knockdown cells failed to exhibit such downregulation of translation-associated signal at sheet-like structures of the ER, which are predominantly confined at perinuclear space (Figure 3D, intensity line plots 4–6). The induction of Fv2E-PERK oligomerization was associated with significant increases in the relative expansion of the ER, which correlated with eIF2α phosphorylation and translation attenuation levels, in the absence of ER stress (Figure S3C).

Brief exposure of tunicamycin-challenged cells to pharmacological translation initiation inhibition (harringtonin or puromycin) completely reverted the ER collapse specifically associated with the abolition of PERK function (Figure 3E). Interestingly, the rescue was associated rather specifically with the blockade of translation initiation and polysome assembly because exposure to cycloheximide, a drug intervening in the elongation step of protein translation and stabilizing polysomes in short-term time frames, was unable to revert the PERK siRNA phenotype (Figure 3E), even under conditions that profoundly inhibit *de novo* protein synthesis.^29^ Our observations support a working model whereby altered regulation of polysome assembly per se, and not dysregulated *de novo* protein synthesis, is a cause of altered ER redistribution in PERK-deficient cells during ER stress.

**Figure 4:**
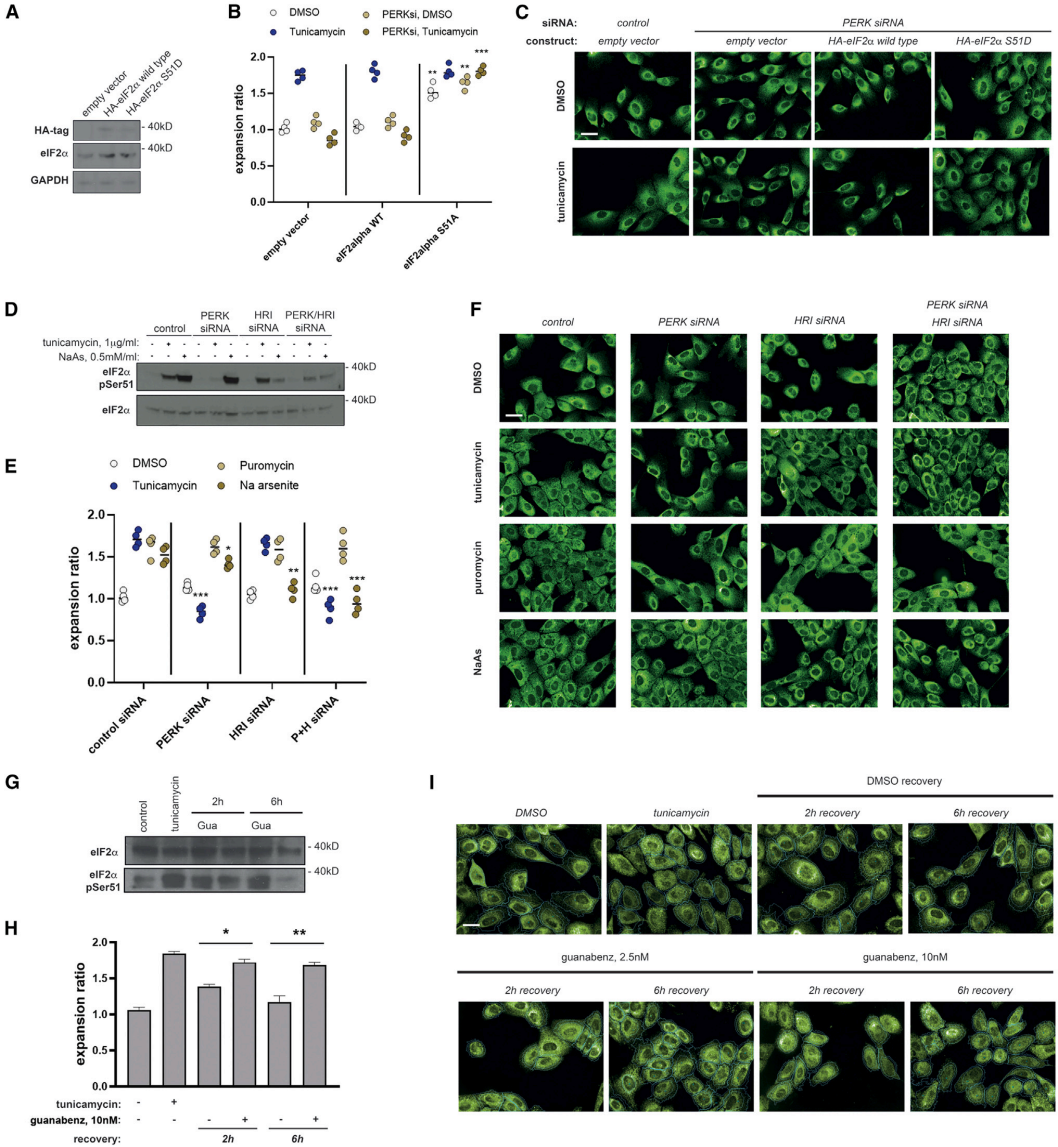
eIF2α phosphorylation is required for PERK-dependent induction of peripheral ER remodeling during ER stress (A–C) Cells stably expressing a phospho-mimicking eIF2α-S51D or the wild-type counterpart (A, western blot analysis) were assessed for peripheral ER remodeling across indicated conditions (B and C). Data are derived from four biological replicates (∼2,000 cells per well). (D–F) Cells transfected with indicated siRNA duplexes were exposed to indicated treatments and analyzed for eIF2α phosphorylation (D) or peripheral ER expansion (E and F). Data are derived from four biological replicates (∼2,000 cells per well). (G–I) MCF10A cells were exposed to tunicamycin for 4 h and then fixed and allowed to recover for indicated times in the presence or absence of guanabenz. Whole-cell extracts were subjected to western blot analysis for indicated markers (G); fixed samples were processed for immunostaining for calreticulin and analyzed for relative peripheral ER content. Data are derived from four biological replicates (∼2,000 cells per well). All scale bars across micrographs represent 15 μm. Statistical significance values from Student’s t tests are indicated as ^∗^*p* < 0.05, ^∗∗^*p* < 0.01, and ^∗∗∗^*p* < 0.005. n.s. *p* > 0.05. Bar graph items show mean values (bar graphs) and standard deviation (error bars); dot plots represent individual replica values, with their average indicated with an horizontal bar.

Cotransfection of an eIF2α S51D phospho-mimetic mutant, but not of a wild-type construct, rescued the ER collapse observed in PERK-depleted, tunicamycin-challenged cells (Figures 4A–4C). We observed moderate but significant increases of ER relative expansion in the absence of ER stress challenge in both wild-type and PERK-depleted cells upon ectopic expression of the phospho-mimicking mutant (Figures 4B and 4C). We performed experiments where we stimulated the activity of an alternative eIF2α kinase during ER stress in PERK-deficient cells. Sodium arsenite specifically stimulates eIF2α phosphorylation through HRI/EIF2AK1 (Figure 4D). Of note, exposure to sodium arsenite led to ER expansion in the absence of ER-targeted drugs in wild-type cells and rescued the ER collapse phenotype of PERK-depleted cells (Figures 4E and 4F). Further supporting the dependence of these phenotypes on eIF2α phosphorylation status, depletion of the HRI kinase prevented sodium-arsenite-derived rescue (Figures 4E and 4F). Finally, we tested the effect of artificially delaying the dynamics of eIF2α phosphorylation using a specific inhibitor of the PPP1R15B phosphatase, guanabenz,^38^ which impairs eIF2α dephosphorylation upon clearance of ER stress (Figure 4G). Exposure to guanabenz delayed the reverse redistribution of the ER associated with ER stress clearance in wild-type MCF10A cells (Figures 4G–4I).

### The integrity of non-centrosomal MTs determines PERK-dependent regulation of ER architecture

**Figure 5:**
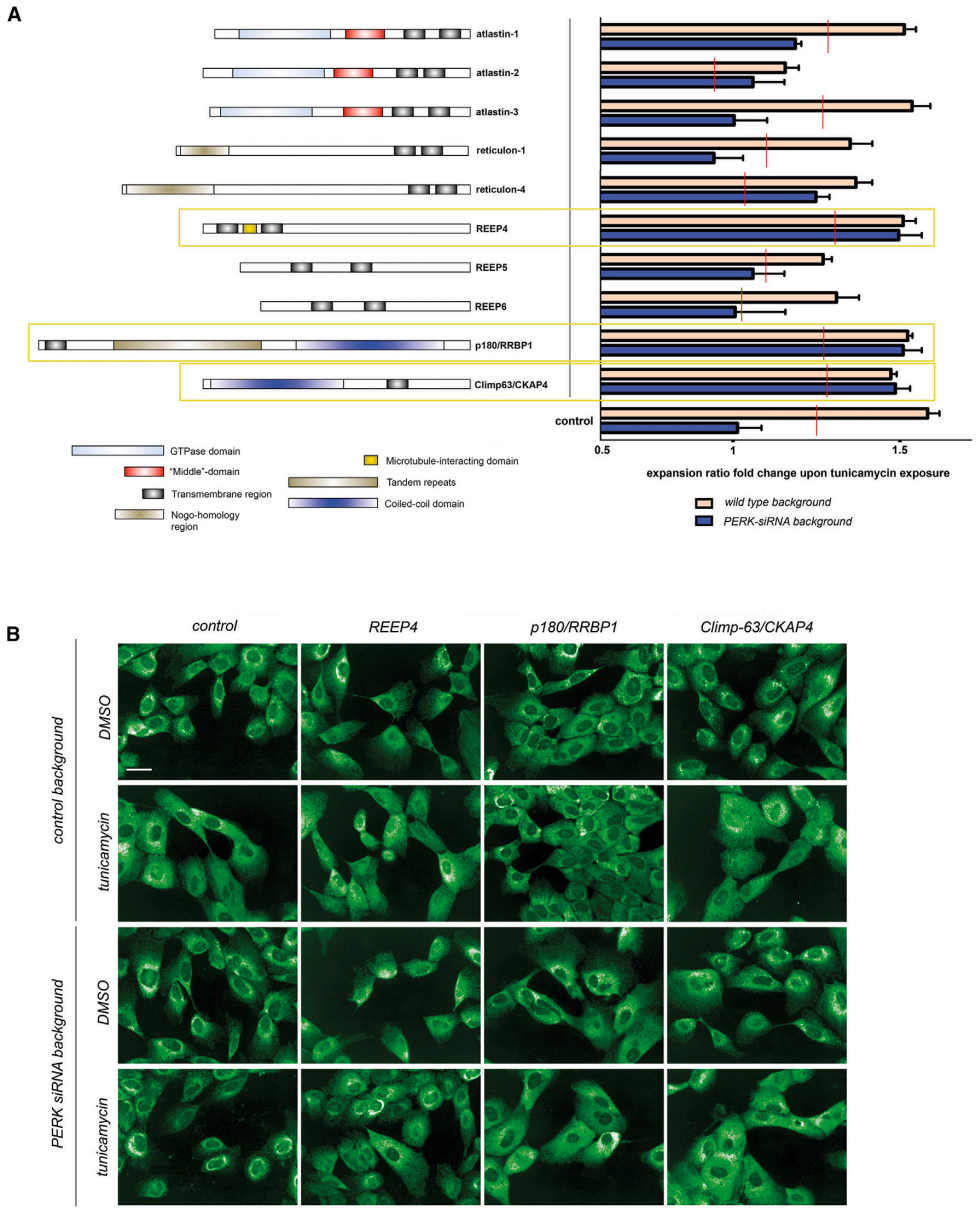
Impaired peripheral ER remodeling in PERK-depleted cells is reverted upon depleting specific ER-shaping proteins that link the ER to the microtubule cytoskeleton (A) The indicated ER shapers (reticulon-4A is the only isoform of RTN4 shown for simplicity) were targeted by siRNA either alone or in combination with an additional siRNA duplex targeting PERK. 48 h later, cell subsets were mock treated with vehicle (DMSO) or exposed for 6 h to tunicamycin. An increase in the ER expansion ratio in tunicamycin-treated subsets, as compared to their DMSO-treated counterparts, is shown for each siRNA group. Red lines indicate the threshold of significance (*p* < 0.05) for the difference between single siRNA treatment and the PERK-ER shaper double siRNA combination. Highlighted targets (yellow frames) exhibit a response comparable to mock-silenced cells and rescue of the phenotype associated with PERK depletion. Data are derived from four biological replicates (∼2,000 cells per well). (B) Representative images of indicated siRNA combinations. Scale bar represents 15 μm.

Using a double siRNA screening approach, we queried a focused list of structural ER “shapers,” known to play specific roles in ER subdomain definition (Figure 5A), to assess whether their inhibition suppressed or enhanced the ER morphogenesis defects observed upon PERK depletion. This small siRNA library was either transfected alone or cotransfected with PERK-targeting siRNA. Finally, these combinations and their corresponding control and PERK siRNA-only controls were exposed to either tunicamycin or vehicle (DMSO) alone. Depletion of REEP4, p180/RRBP1, and Climp63/CKAP4 rescued the PERK siRNA phenotype of ER collapse upon tunicamycin exposure (highlighted in Figures 5A, 5B, and S4A). siRNAs targeting these proteins were effective and did not affect the levels of PERK mRNA (Figure S4B). This rescue did not correlate with evident alterations of protein translation, as inferred from PMY staining (Figure S4C). Curiously, these hits are mostly conserved in higher metazoans, and orthologs are not found across clades where EIF2AK3/PERK is absent,^39^ suggesting a co-evolved functional relationship (Figure S4D).

Because REEP4, p180/RRBP1, and Climp63/CKAP4 share the property of anchoring the ER to the MT network,^24^^,^^27^^,^^40^ we tested the involvement of MT stability and organization in the phenotypes associated with PERK deficiency by disrupting the MT network with nocodazole. Nocodazole treatment reverted the observed ER collapse associated with tunicamycin challenge in PERK-depleted cells (Figure S4E). This effect is not dependent on translation activity per se because nocodazole-treated cells are still competent for ribosome engagement in protein synthesis at the times and concentrations tested (Figure S5A). We interpret that ER collapse associated with PERK deficiency is driven by dysregulated interactions between specific ER shapers and MTs.

**Figure 6:**
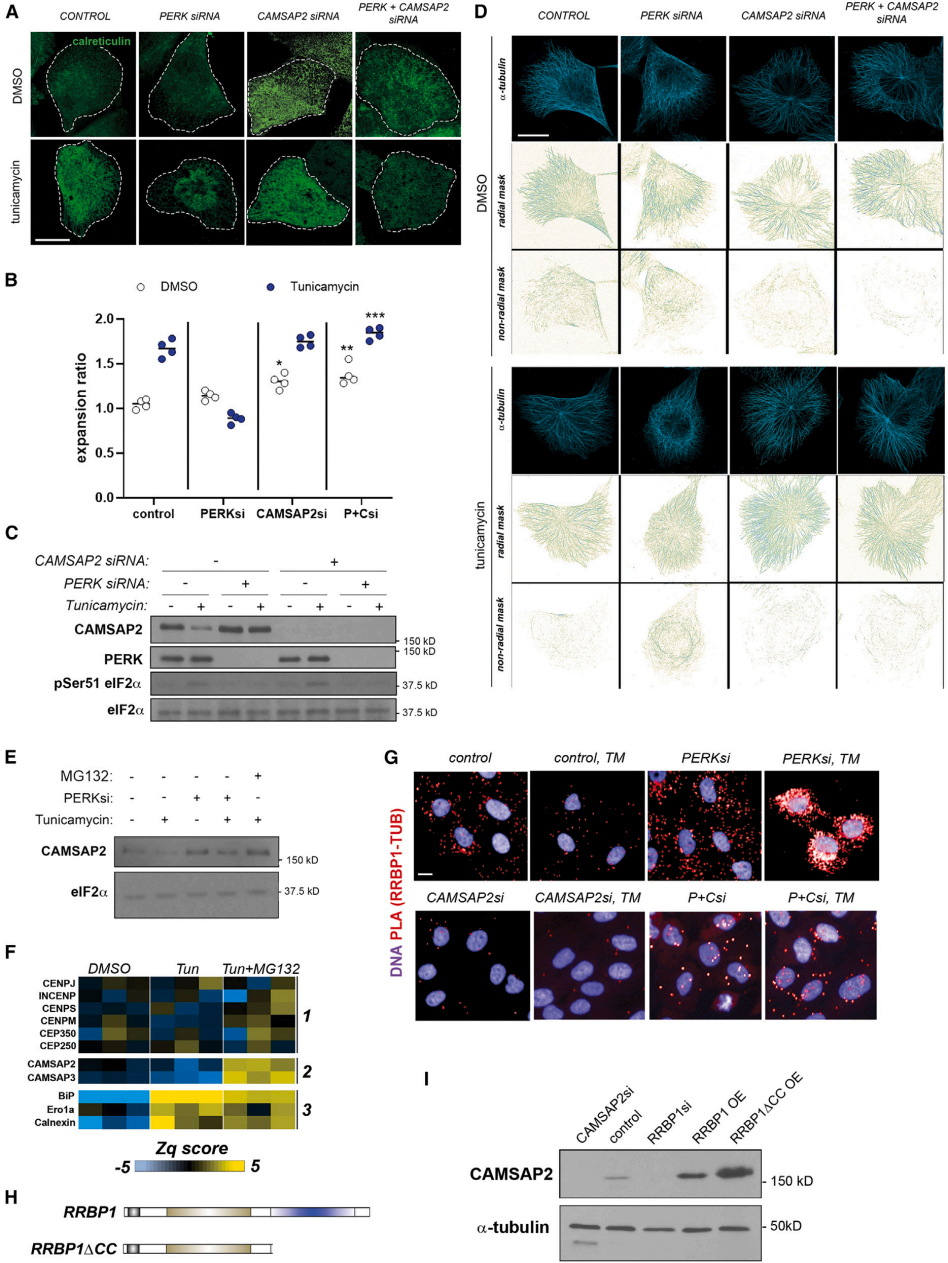
Non-centrosomal microtubules are involved in the phenotype of impaired peripheral ER remodeling associated with PERK depletion (A–C) MCF10As were reverse transfected with indicated siRNA combinations, exposed to indicated small-compound treatments, and analyzed by quantitative imaging (A and B) or western blot (C). Data in (B) are derived from four biological replicates (∼2,000 cells per well). (D) Analysis of radial and non-radial microtubule signals from MCF10A cells subjected to indicated siRNA and small-compound treatments. (E) MCF10A cells, either mock or PERKsi transfected, were exposed to 5 μg/mL tunicamycin for 6 h and assayed by western blot for the indicated markers. Where indicated, cells were simultaneously exposed to MG-132 proteasome inhibitor (1 μM). (F) MCF10A cells were exposed to DMSO or 5 μg/mL tunicamycin alone for 6 h or simultaneously exposed to tunicamycin and the MG-132 proteasome inhibitor (1 μM) and analyzed by quantitative proteomics (see also Table S2). A heatmap of Zq values (spectra, peptide, and protein values are integrated, and their deviation from average values obtained across samples is then expressed as normalized to the variability for each sample; see STAR Methods) of all three replicas per condition is shown. Three protein groups are highlighted: centrosomal proteins (1), non-centrosomal microtubule proteins (2), and canonical ER stress markers (3). (G) Spinning disk confocal images of proximity ligation assay samples assayed for RRBP1-αtubulin interaction (red signal) across indicated conditions. ER stress induction treatments where indicated were 5 μg/mL tunicamycin for 6 h (see also Figures S5H and S5I for further control conditions and quantitation). (H) Architecture of lentiviral p180 expression constructs assayed in (I) and Figures S5H and S5I. (I) MCF10A cells reverse transfected with indicated siRNAs or transduced with indicated lentiviral expression constructs (OE) were analyzed by western blotting as indicated. All scale bars across micrographs indicate 10 μm. Statistical significance values from Student’s t tests are indicated as ^∗^*p* < 0.05, ^∗∗^*p* < 0.01, and ^∗∗∗^*p* < 0.005. n.s. *p* > 0.05. The dot plot in (B) represents individual replica values, with their average indicated with an horizontal bar.

MTs can either be nucleated at centrosomes or form non-centrosomal MT bundles.^41^ We first assessed whether centrosomal MTs determined ER cell distribution and architecture by depleting centrosome structures from MCF10A cells using the Polo-like kinase 4 (PLK4) inhibitor centrinone.^42^ Depletion of MTs nucleated at the centrosome did not rescue the ER expansion defect associated with PERK depletion (Figures S5B and S5C). Next, we knocked down CAMSAP2, a well-established minus-end stabilizer of non-centrosomal MTs^41^^,^^43^ (Figure 6A). Cells deficient for stabilization of non-centrosomal MTs alone exhibited a moderate phenotype of increased peripheral ER cistern-like structures (Figures 6A and 6B). Importantly, the knockdown of CAMSAP2 in PERK-deficient cells fully rescued the phenotype of impaired ER peripheral distribution associated with PERK depletion (Figures 6A and 6B). Of note, CAMSAP2 depletion attenuated the partial tunicamycin-induced loss of cell viability in PERK-deficient cells, albeit viability in DMSO-treated double-knockdown cells was also slightly affected (Figure S5D). In accordance with the increased peripheral extension of sheet-like structures, CAMSAP2-depleted cells showed higher signal density for RRBP1 immunostaining in the cell periphery (Figure S5E).

We appreciated in previous experiments that acute ER stress induction was associated with the presence of prominent, discrete MT nucleation centers in wild-type cells, whereas PERK-depleted cells did not exhibit such features (see Figure S4D, white arrowheads). To study this phenomenon in more detail, we obtained MT image sets using STED microscopy from cells exposed to acute ER stress either transfected with scrambled siRNA or depleted for PERK, CAMSAP2, or both proteins simultaneously (Figure 6C; see also Figure 6D). As expected, the depletion of CAMSAP2 alone led to a relative decrease in non-radial MT structures, supporting a loss of non-centrosomal MTs (DMSO). Wild-type cells also exhibited a decrease in non-radial MT structures when exposed to acute ER stress, suggesting that ER stress suppresses non-centrosomal MT polymerization/stability. Of note, this phenotype correlated with a significant decrease in CAMSAP2 total protein of ∼50% (see Figure 6D). However, this ER-stress-induced decrease in non-centrosomal MTs and its central regulator, CAMSAP2, was not displayed by cells depleted from PERK (Figures 6C and 6D). These observations support that PERK suppresses non-centrosomal MT polymerization/stabilization. Finally, cells depleted of both PERK and CAMSAP2 had very few non-centrosomal MTs, phenocopying CAMSAP2 knockdown, suggesting that PERK-driven suppression of non-centrosomal MTs is upstream of CAMSAP2.

RT-qPCR analysis did not provide support for a transcriptional mechanism driving the observed modulation of CAMSAP2 levels and non-centrosomal MTs by ER stress (Figure S5F). Because our previous studies suggested that the protein synthesis ratio per se is not a major relevant mechanism driving the phenotypes of altered ER expansion (see Figure 3E), we hypothesized that differential protein turnover could be a molecular explanation for the observed sensitivity of CAMSAP2 protein levels and non-centrosomal MT density to ER stress. Supporting protein stability as a mechanism for the observed decrease in CAMSAP2 levels upon ER stress induction, exposure to proteasome inhibitor MG-132 increased basal CAMSAP2 levels and blunted their reduction associated with tunicamycin treatment (Figure 6E). Quantitative proteomics corroborated that ER stress reduces the levels of non-centrosomal MT proteins in a proteasomal-degradation-dependent manner (Figure 6F).^25^^,^^44^

We hypothesized that ER-MT physical anchoring, dependent on a non-centrosomal, CAMSAP2-stabilized MT population, is a central molecular event underlying the observed phenotypes. Recent studies suggest that specific posttranslational modifications (PTMs) targeting MTs could modulate the anchoring of the ER and MTs.^28^ Western blot analysis failed to reveal significant changes in MT glutamylation upon CAMSAP2 knockdown, albeit a reduction in MT acetylation was observed (Figure S5G). Notably, RRBP1 levels were unaffected by CAMSAP2 knockdown; our own proteomics analyses, as well as previous literature,^29^ support that acute ER stress does not reduce RRBP1 levels. To monitor ER-MT anchoring in our system, we established a proximity ligation assay (PLA) reporting an RRBBP1/p180-αtubulin interaction (Figures S5H, S5I, and 6G). This interaction was significantly diminished upon ER stress induction, as well as upon CAMSAP2 knockdown (Figures 6G and S5I). PERK knockdown increased ER-MT anchoring and induced a perinuclear accumulation pattern upon tunicamycin exposure; these patterns were abolished upon simultaneous knockdown of CAMSAP2 (Figures 6G and S5I). Of note, this ER-MT physical anchoring is sensitive to translation initiation blockade (Figures S5H and S5I).

ER-MT anchoring could modulate MT stability.^44^^,^^45^ While RRBP1/p180 knockdown reduced CAMSAP2 levels as compared to mock-transfected cells, the overexpression of a construct bearing the MT-binding domain of RRBP1/p180, known to strengthen ER-MT anchoring,^24^^,^^45^ partly stabilized CAMSAP2 (Figures 6H and 6I; see also Figures S5H and S5I). Our data suggest that ER anchoring through a specific subset of MTs and specialized tethers, such as RRBP1, governs the architecture of the ER itself, as well as the stability of that same population of MTs, conforming a feedback mechanism that supports and consolidates a coordinated phenotype for the ER and MT networks according to different conditions.

### Disruption of PERK kinase activity impacts cell protrusiveness and motility through mechanisms dependent on non-centrosomal MTs

**Figure 7:**
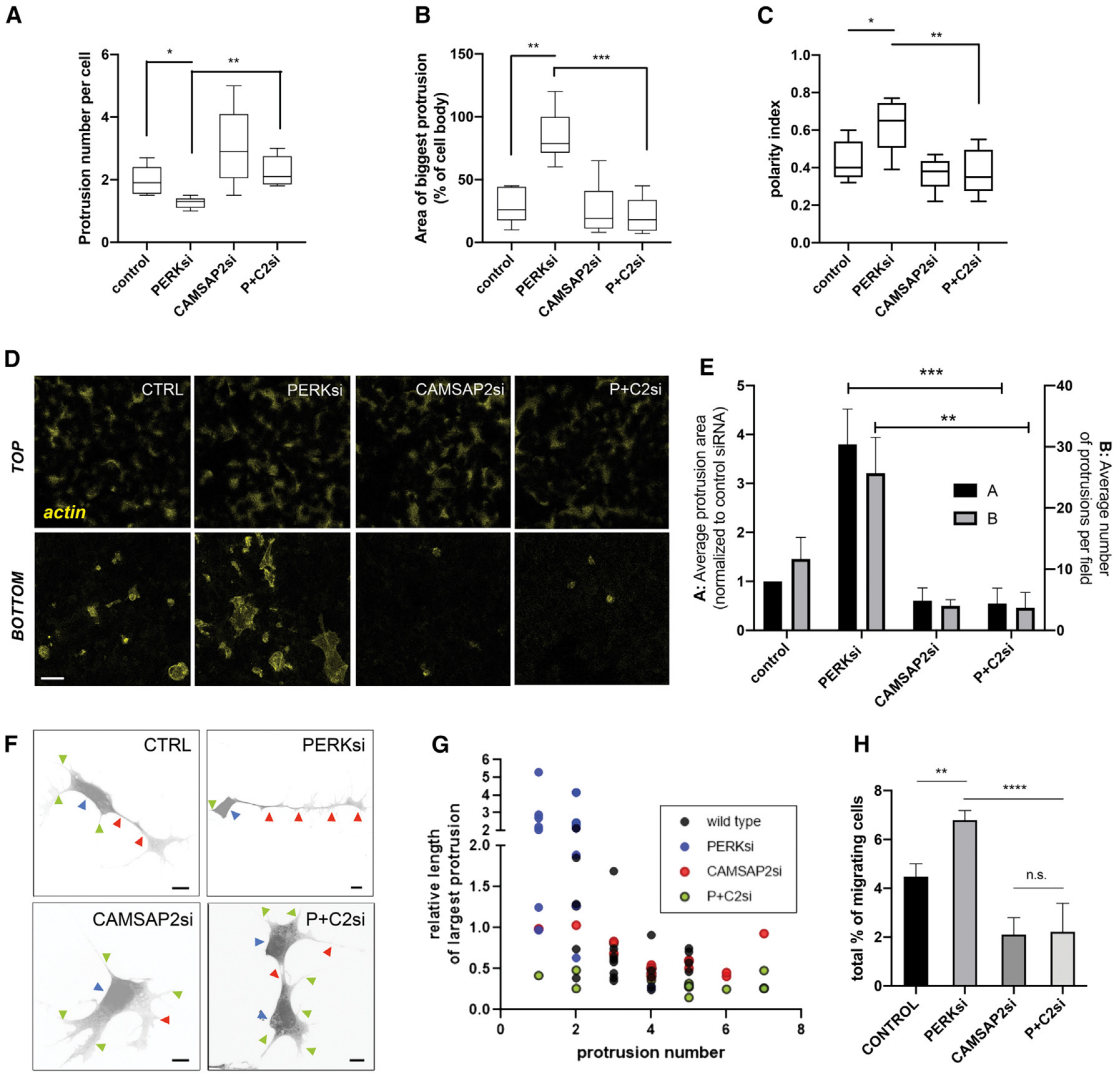
PERK depletion impacts cell protrusiveness and polarized migration in a manner dependent on non-centrosomal microtubules (A–C) Features indicated were extracted from a minimum of 25 cytoplasm-counterstained MCF10A cell confocal images across indicated conditions. (D and E) MCF10As subjected to indicated siRNA treatments were allowed to protrude through transwell pore membranes for 4 h and then fixed and stained for actin and imaged. (E) Indicated parameters were computed from 10 independent fields per condition from 3 biological replicates. Scale bar represents 5 μm. (F and G) SH-Sy5y neuroblasts transfected with indicated esiRNAs were stimulated for neural differentiation (see STAR Methods). Cell soma (blue arrowheads), the longest protrusion (red arrowheads), and secondary protrusions (green arrowheads) are highlighted. All scale bars represent 10 μm. (G) The number of protrusions per cell and the length of the longest protrusion relative to the perimeter of the cell soma are plotted for each condition (*n* = 20 from 2 biological replicates). (H) MCF10As subjected to indicated siRNA treatments were allowed to migrate through 3D collagen matrices and imaged at plate bottom and three consecutive optical sections 50 μm apart. The percentage of cells migrated from total cell count is indicated. Data are derived from six biological replicates. Statistical significance values from Student’s t tests are indicated as ^∗^*p* < 0.05, ^∗∗^*p* < 0.01, and ^∗∗∗^*p* < 0.005. n.s. *p* > 0.05. Bar graph items show mean values (bar graphs) and standard deviation (error bars); dot plot in (G) represents individual cell values. Boxplots indicate data range (error bars), confidence interval (box edges), and average values (central line).

Non-centrosomal MT arrays can regulate cell protrusiveness and migration.^41^^,^^43^ Depletion of PERK kinase in cells led to increased protrusion size but reduced protrusion number and increased polarity as compared to wild-type cells (Figures 7A–7C). These observations are in agreement with an interpretation that PERK activity could regulate cell polarity by suppressing the polymerization of non-centrosomal MTs, which contribute to cell protrusiveness.^43^ Increased protrusiveness was not observed in PERK-deficient cells simultaneously depleted for CAMSAP2 (Figures 7A–7C).

To further assess the apparent directional protrusiveness phenotype of PERK-depleted cells, we performed transwell protrusion/migration assays. We observed a higher number of protrusions passing through membrane pores when depleting cells for the PERK kinase using siRNA, as well as upon exposure to the PERK allosteric kinase inhibitor (Figure 7D; see also Figure S6A). Importantly, this increase in protrusiveness was abrogated by the simultaneous depletion of the non-centrosomal minus-end stabilizer CAMSAP2 (Figures 7D and 7E). Increased polarized protrusiveness associated with PERK depletion was also observed during neurite formation in neuroblasts (Figure 7F): PERK-deficient cells exhibited very long unique protrusions as compared to wild-type cells. Simultaneous depletion of CAMSAP2 reverted these phenotypes. In agreement with previous studies^46^^,^^47^ and our previous observations (see Figures 6D and 6E), inhibition of proteasomal turnover was associated with an increased CAMSAP2-dependent elongation of polarized protrusions in neuroblasts (Figure S6B).

We studied the impact of intervening PERK signaling on three-dimensional (3D) cell behavior. PERK inhibition enhanced cell migration through a 3D collagen matrix, and this phenotype was inhibited upon depletion of the non-centrosomal MT stabilizer CAMSAP2 (Figure 7H). In accordance, cells plated on soft collagen matrices exhibited long protrusions (Figure S6C). Of note, depletion of the ER-MT linker RRBP1/p180 also reduced the increase in elongated protrusions and abrogated the increase in 3D cell migration (Figures S6C and S6D). Artificial PERK activation in the synthetic Fv2E-PERK model by exposure to the homodimerizer AP20178 had an opposite effect on 3D cell migration, further supporting a role for PERK-dependent signaling on the regulation of cell motility (Figure S6E). We propose a model whereby ER-MT reciprocal regulation through PERK-dependent translation control has a dual impact on both regulated ER remodeling and non-centrosomal MT-dependent cell polarity and migration.

## Discussion

Eukaryotes evolved systems that gauge ER physical integrity and function and coordinate different responses in the cell to primarily adapt its function, collectively termed the UPR. Here, we contribute evidence that UPR^ER^/PERK-dependent control of protein translation modulates the reciprocal regulation of ER and non-centrosomal MTs to both drive ER remodeling during acute ER stress and regulate non-centrosomal MT-dependent cell protrusiveness and polarity.

Both ribosome association and MT cytoskeleton integrity can affect ER architecture, as well as the localization of ER shapers.^29^ We demonstrate that this relationship is controlled by the PERK kinase through eIF2α-phosphorylation-mediated translation shutdown. ER architecture is tightly regulated by different shaper proteins, capable of defining different ER membrane domains.^2^ The targeting of ER-shaping proteins must be controlled to allow for the dynamic remodeling of these membrane structures for cell adaptation to different functional states, and different mechanisms have been suggested, including PTMs and discrimination of differential membrane curvature.^27^^,^^48^^,^^49^^,^^50^ Our results support that the coordination of the dynamics of polysomes (presumably translocon-engaged units), MTs, and specific ER-anchored proteins is pivotal. Further, our observations and previous literature^2^^,^^29^ suggest that polysome physical assembly per se, and not protein synthesis throughput, is a key component in this system. A possibility explaining this multiple-component requirement is that ribosome and MT bindings by ER-MT tethers are events potentially mutually influencing each other, as suggested by other studies^28^^,^^51^^,^^52^^,^^53^; further structural details of these interactions might shed light on their regulation. Further supporting the specific effect of the physical presence of translation-competent units, as opposed to protein translation output, in modulating the ER-MT relationship, the overexpression of full-length translocon-component Sec61β induces an MT phenotype reminiscent of exacerbated ER-MT anchoring.^53^

The levels and modulation of ER-MT tethers can have an impact on the architecture of the MT network.^24^^,^^27^ We failed to record changes in MT glutamylation upon either ER stress induction or CAMSAP2 knockdown (Figure S5G); while these PTMs have been recently reported to contribute to the specificity of ER-MT tethering mechanisms,^28^ it remains to be explored whether and how they play a role in ER-stress-induced ER remodeling. Our studies support that ER-MT tethering also specifically controls non-centrosomal MTs and their regulator CAMSAP2 through mechanisms involving controlled proteasomal turnover. These observations could have a relationship with recent studies indicating proteasomal turnover as a means to control the MT network during cell stress.^54^ Non-centrosomal MT nucleators may be differentially targeted for ubiquitination according to the anchoring state of this MT population.

Physical ER expansion and shape remodeling is a relevant adaptive event in cells with compromised ER function (i.e., ER stress)^5^ and correlates with other adaptive responses such as the upregulation of protein maturation machinery.^55^ PERK-dependent coupling of attenuation of client protein load and ER expansion and remodeling would ensure matching of both adaptive ER volume increase and protective translation attenuation. ER membrane expansion might be partly enabled by this coupling, which would limit the density of ER-membrane-inserted proteins. We do not know the precise relationship of our findings with mechanisms driving ER membrane biogenesis.^5^^,^^56^ We have observed that the depletion of non-centrosomal MTs attenuates the relative sensitivity of PERK-deficient cells to acute ER stress, although CAMSAP2 depletion per se seems to have an impact on ER homeostasis (see Figure S5D). Future studies will be required to characterize in detail how architectural changes in the ER impact different outputs of ER function. Previous studies suggested a specialization of ER-associated MT pools.^22^ An emerging major role of non-centrosomal MTs, as suggested by studies manipulating this MT subpool, is the onset and maintenance of cell polarity and protrusiveness across cell types.^41^^,^^43^ Importantly, cell polarity and protrusiveness are linked to organelle dynamics and trafficking through mechanisms that rely on the control of organelle architecture and couple them to *de novo* membrane synthesis.^57^^,^^58^ It is intuitive that their coupling to ER stress surveillance is convenient to ensure cell homeostasis across functional states.

Neuron differentiation and the control of neurite outgrowth and axon stabilization are largely determined by the fine regulation of ER architecture, MT organization, and their reciprocal crosstalk,^45^^,^^59^^,^^60^ together with local protein translation, and challenging ER homeostasis can compromise this specialized form of cell protrusion stabilization.^61^ Non-centrosomal MTs appear to be specifically involved in these mechanisms.^45^^,^^62^^,^^63^ Because eIF2α phosphorylation and PERK activity modulate memory stability and learning,^64^^,^^65^^,^^66^ it will be interesting to study the relevance of our findings in these contexts. Future studies focusing on the impact of these mechanisms on directional vesicle trafficking and *de novo* membrane formation, which seem to be relevant for protrusion formation, may also shed light on these questions.^58^ PERK activity has also been studied in the context of tumorigenesis and its complex impact on tumor cell survival and tissue organization.^35^^,^^67^^,^^68^^,^^69^ Recent studies support a role for CAMSAP2 in tumor cell invasiveness.^70^ The impact of intervening PERK signaling on tumor cells is highly contextual and simultaneously affects different aspects of tumor cell biology: cell survival and adaptation to nutrient deprivation and adverse environment, adhesion signaling, and accommodation of altered secretory phenotypes. Our results contribute an additional perspective to this complex picture and suggest novel opportunities for synergistic intervention of tumor cell biology by shutting down PERK-dependent prosurvival signaling (autophagy and reactive oxygen species [ROS] management) and cell invasiveness.

### Limitations of the study

All experiments using automated spinning disk microscopy were, by definition, acquired at the same focal plane across all samples. STED experiments were primarily aimed at obtaining high-resolution images of the collapsed ER structure: this might explain a slight plane height difference across images, together with the inherent higher x-y resolution. Further, PERK-deficient cells exposed to tunicamycin also exhibited a less regular nuclear boundary profile, which might contribute to a different appearance of the nuclear region. Our findings are reproduced across different cell lines (Figures S1 and 7) but are yet to be explored in an *in vivo* setting. We have studied the mechanistic relationship between non-centrosomal MTs and ER anchoring through RRBP1, but at present, we do not know if the phenotype reversion we observed upon knocking down other ER shapers is based on the same precise molecular mechanisms.

## Resource availability

### Lead contact

Requests for further information and resources should be directed to and will be fulfilled by the lead contact, Miguel Sánchez-Álvarez (msalvarez@iib.csic.es).

### Materials availability

All unique/stable reagents generated in this study are available from the lead contact with a completed materials transfer agreement.

### Data and code availability

• Data availability: the mass spectrometry proteomics data (Figure 6F) have been deposited to the ProteomeXchange Consortium via the PRIDE partner repository with the dataset identifier PRIDE: PXD057012. High-content image data have been deposited at Zenodo with unique identifier Zenodo: https://doi,org/10.5281/zenodo.14041811.
• Code availability: our automated image analysis pipeline is available as a supplemental ZIP file (Data S1).

## Acknowledgments

Light microscopy and dynamic imaging/ICTS-ReDib at CNIC is supported by MCIN/AEI/10.13039/501100011033 and FEDER “Una manera de hacer Europa” (#ICTS-2018-04-CNIC-16; Madrid, Spain). Amine Sadok and Faraz Mardakheh (former researchers at ICR, London, UK) provided expert advice and assistance with collagen migration experiments. C.B. and H.S. have been beneficiaries of the Wellcome Trust Career Development Fellowship program. Funding support at the C.B. lab was received from the Cancer Research UK (CRUK) Programme Foundation Award (C37275/A20146) and the Stand Up to Cancer campaign. M.S.-A. was a fellow of the COFUND-IPP program (CNIC); is a recipient of grants from the Spanish Ministerio de Ciencia e Innovación (MICINN; RYC2020-029690-I and PID2021-128106NA-I00) and the 10.13039/501100002704Scientific Foundation, Spanish Association Against Cancer (LAB AECC 2024 grant LABAE246690SANC); and is supported by consolidation grant CNS2023-144831, sponsored by Ministerio de Ciencia, Innovación y Universidades (MICIU)/AEI/10.13039/501100011033 and European Union NextGenerationEU/PRTR. The M.A.d.P. lab is sponsored by Spanish Ministerio de Ciencia e Innovación (MCNU; PID2020-118658RB-I00, SAF2017-83130-R, and BFU2016-81912-REDC); the Comunidad Autónoma de Madrid/FEDER, Spain (ref. S2018/NMT4443; Actividades de I+D entre Grupos de Investigación en Tecnologías); Obra Social La Caixa (AtheroConvergence-HR20-00075); and the 10.13039/100008666Fundació la Marató de TV3 (385/C/2019). The CNIC is supported by the 10.13039/501100004587Instituto de Salud Carlos III (ISCIII), the Ministerio de Ciencia, Innovación y Universidades (MICIU), and the Pro CNIC Foundation and is a Severo Ochoa Center of Excellence (grant CEX2020-001041-S funded by MICIU/AEI/10.13039/501100011033). The M.S-A. laboratory is a member of the RER-CSIC rare disease research network.

## Author contributions

M.S.-A. and C.B. conceived and designed the study. H.S. provided image analysis tools for the initial screen. M.S.-A., F.L., G.F., L.A., and M.C.-M. performed experimental work and analyzed the data. J.A.L. and J.V. performed quantitative proteomics. P.P.-V. and M.A.-G. acquired relevant preliminary results. M.A.d.P. provided resources. M.S.-A. and C.B. wrote the paper.

## Declaration of interests

The authors have no competing interests to declare.

## STAR★Methods

### Key resources table

**Table:** 

REAGENT or RESOURCE	SOURCE	IDENTIFIER
**Antibodies**		

anti-Calreticulin	abCam	Cat. Num. ab2907; RRID:AB_303402
anti-eIF2α, phosphoserine 51 (immunofluorescence, western blotting)	Enzo Biosciences	Cat. Num. BML-SA405; RRID:AB_2097934
anti-eIF2α, phosphoserine 51 (western blotting). (D9G8) XP Rabbit mAb	Cell Signaling Technologies	Cat. Num. #3398; RRID:AB_2096481
total eIF2α (D7D3) XP Rabbit mAb	Cell Signaling Technologies	Cat. Num. #5324; RRID:AB_10692650
anti-SREBP1a/c, 2A4 clone	abCam	ab3259; RRID:AB_303650
anti-ATF-4 (D4B8) Rabbit mAb	Cell Signaling Technologies	Cat. Num. #11815; RRID:AB_2616025
anti-puromycylated polysomes	Developmental Studies Hybridoma Bank repository	Cat. Num. PMY-24B; RRID:AB_2619605
anti-HA tag (12CA5 clone)	Roche	Cat. Num. 45-11583816001; RRID:AB_1074940
Anti-RRBP1	Proteintech	Cat. Num. 22015-1-AP; RRID:AB_2878971
anti-αtubulin, DM1A clone (immunofluorescence, western blot)	Sigma	Cat. Num. T6199; RRID:AB_477583
anti-αtubulin, YOL1/34 clone (rat) (immunofluorescence)	GeneTex	Cat. Num. GTX26161; RRID:AB_385177
anti-CAMSAP2	Proteintech	Cat.Num. 17880-1-AP; RRID:AB_2068826
Anti-PERK (D11A8) Rabbit mAb	Cell Signaling Technologies	Cat. Num. #5683; RRID:AB_10841299
anti-acetyl-αtubulin 6-11B-1 clone	Sigma	Cat. Num. MABT868; RRID:AB_2819178
anti-polyglutamylated-αtubulin (GT335)	Adipogen	Cat. Num. AG-20B-0020-C100; RRID:AB_2490210
Goat anti-Rabbit IgG (H + L) Cross-Adsorbed Secondary Antibody, Alexa Fluor 488	Thermo Scientific	Cat. Num. A-11008; RRID:AB_143165
Goat anti-Rabbit IgG (H + L) Cross-Adsorbed Secondary Antibody, Alexa Fluor 568	Thermo Scientific	Cat. Num. A-11011; RRID:AB_143157
Goat anti-Mouse IgG (H + L) Cross-Adsorbed Secondary Antibody, Alexa Fluor 647	Thermo Scientific	Cat. Num. A-21235; RRID:AB_2535804
Goat anti-Rat IgG (H + L) Cross-Adsorbed Secondary Antibody, Alexa Fluor 568	Thermo Scientific	Cat. Num. A-11077; RRID:AB_141874
Peroxidase AffiniPure Goat Anti-Rabbit IgG (H + L)	Jackson ImmunoResearch	Cat. Num. 111-035-003; RRID:AB_2313567
Peroxidase AffiniPure Goat Anti-Mouse IgG (H + L)	Jackson ImmunoResearch	Cat. Num. 115-035-003; RRID:AB_10015289

**Bacterial and virus strains**		

DH5α, chemocompetent E. coli	CNIC core services	N/A
STBL3, chemocompetent E. coli	CNIC core services	N/A

**Chemicals, peptides, and recombinant proteins**		

insulin	Sigma	Cat. Num. I-1882
hydrocortisone	Sigma	Cat. Num. H-0888
cholera toxin	Sigma	Cat. Num. C-8052
epidermal growth factor	Preprotech	Cat. Num. AF-100-15
Lipofectamine RNAiMAX	Invitrogen-Thermo Scientific	Cat. Num. 13778075
Lipofectamine 3000	Invitrogen-Thermo Scientific	Cat. Num. L3000008
tunicamycin	Sigma	Cat. Num. T7765
MG-132	Sigma	Cat. Num. 474790
sodium arsenite	Sigma	Cat. Num. S-7400
cycloheximide	Sigma	Cat. Num. 239765
nocodazole	Sigma	Cat. Num. M-1404
guanabenz	Sigma	Cat. Num. 370625
Hoescht 33342	Sigma	Cat. Num. B-2265
thapsigargin	Santa Cruz	Cat. Num. sc-24017
propargylcholine	Jena Biosciences	Cat. Num. CLK-066
AZDye647-Azide (Alexa 647-azide conjugate)	Jena Biosciences	Cat. Num. CLK-1299
Cell Tracker Orange CMRA	Thermo Scientific	Cat. Num. C34551
3-(4,5-dimethylthiazol-2-yl)-2,5-diphenyltetrazolium bromide (MTT)	Sigma-Merck	Cat. Num. 475989
GSK2606414 PERK inhibitor	Tocris	Cat. Num. 5107/10
Bovine serum albumin, endotoxin-free	NZYTech	Cat. Num. 11-523
(Homo)harringtonin	Tocris	Cat. Num. 1416/10
AP20187 homodimerizer	Selleckchem	Cat. Num. S8487
centrinone	R&D systems	Cat. Num. 5687/10
Cytiva(Amersham) Hybond-P PVDF membrane	Amersham-Fisher Scientific	Cat. Num. 15259894
Cytiva(Amersham) ECL Detection Reagent	Amersham-Fisher Scientific	Cat. Num. RPN3004
Rat Tail Type-1 collagen	Corning	354236
Methanol-free, 16% paraformaldehyde-PBS	ThermoScientific	Cat. Num. 043368.9M
Trypsin Gold, mass spectrometry grade	Promega	Cat. Num. V5280
Deoxynucleotides (100mM stock)	Thermo Scientific	Cat. Num. 10297-018
MuMLV 5x retrotranscriptase	Promega	Cat. Num. M531A
Random primers	Promega	Cat. Num. C1181
All cloning enzymes (T4 ligase, restriction enzymes)	NEB	N/A
*trans*-retinoic acid	Sigma	Cat. Num. 554720
BDNF	Sigma	Cat. Num. SRP3014
ER-Tracker Green (BODIPY FL Glibenclamide), for live-cell imaging	Molecular Probes, ThermoScientific	E34251
puromycin	Sigma	Cat. Num. P7255
Gibco Dulbecco’s Modified Eagle Medium: Nutrient Mixture F12 (DMEM F12), GlutaMax	Thermo Scientific	Cat. Num. 10565018
Gibco Dulbecco’s Modified Eagle Medium (DMEM), GlutaMax	Thermo Scientific	Cat. Num. 10566016
Gibco Dulbecco’s Modified Eagle Medium: Nutrient Mixture F12, phenol red-free	Thermo Scientific	Cat. Num. 21041025
Gibco Horse Serum	Thermo Scientific	Cat. Num. 16050122
Gibco Fetal Bovine Serum	Thermo Scientific	Cat. Num. A5256701
Gibco Penicillin/Streptomycin x100	Thermo Scientific	Cat. Num. 15140122
Gibco L-Glutamine 200mM	Thermo Scientific	Cat. Num. A2916801
Gibco Fungizone	Thermo Scientific	Cat. Num. 15290026
Plasmocin, prophylactic	Ibian Technologies	Cat. Num. ant-mpp
Gibco non-essential amino acid mixture	Thermo Scientific	Cat. Num. 11140050
Gibco β-mercaptoethanol	Thermo Scientific	Cat. Num. 21985023

**Critical commercial assays**		

QIAGEN MaxiPrep kit	QIAGEN	Cat. Num. 12163
RNAeasy RNA purification kit	QIAGEN	Cat. Num. 74104
iQ SYBR Green Supermix	BioRad	Cat. Num. 1708882
Taq 2X MasterMix	New England Biolabs	Cat. Num. M0270
TMT 11plex reagents	Thermo Fisher	Cat. Num. A34808
High pH Reversed-Phase Peptide Fractionation Kit	Pierce	Cat. Num. 84868
CLICK chemistry reaction kit	Jena Biosciences	Cat. Num. CLK-074
DuoLink Orange commercial kit	Sigma-Aldrich	Cat. Num. DUO92102
Pall Life Sciences Nanosep 30k Omega Membrane Ultrafiltration UF Centrifugal Device Spin Filter Sample Volume 50 To 500ul	Pall Life Sciences	Cat. Num. PLS2386
Waters Corporation Oasis HLB Online Column	Fisher Scientific	Cat. Num. 186002041
Hanging Millipore Transwell inserts, 24-well plate	Merck Millipore	Cat. Num. PITP01250

**Deposited data**		

ProteomeXchange Consortium via the PRIDE partner repository.	This study	PXD057012
High-content screening data	This study	https://doi.org/10.5281/zenodo.14041811

**Experimental models: Cell lines**		

MCF10A cells	Claire Isacke (ICR, UK)	N/A; RRID:CVCL_0598
MCF10A AT cells	Claire Isacke (ICR, UK)	N/A; RRID:CVCL_5555
HeLa cells	ATCC	Cat. Num. CCL-2; RRID:CVCL_0030
MDA-MB231 cells	ATCC	Cat. Num. HTB-26; RRID:CVCL_0062
Sh-Sy5y cells	Sergio Casas Tintó (Instituto Carlos III, Spain)	N/A; RRID:CVCL_0019
MCF10A/Fv2E-PERK cells	Julio Aguirre-Ghiso (Mount Sinai Hospital, USA)	Avivar-Valderas et al.^34^
MCF10A/PERKΔC cells	Julio Aguirre-Ghiso (Mount Sinai Hospital, USA)	Sequeira et al.^35^

**Oligonucleotides**		

PERK (siRNA pool)	Dharmacon	M-004883-03
PERK (siRNA)	Dharmacon	J-004883-09
PERK (siRNA)	Dharmacon	J-004883-10
PERK (siRNA)	Dharmacon	J-004883-11
PERK (siRNA)	Dharmacon	J-004883-12
PERK (siRNA	Dharmacon	L-004883-00
IRE1 (siRNA pool)	Dharmacon	M-004951-02
ATF6 (siRNA pool)	Dharmacon	M-009917-01
ATL1 (siRNA pool)	Dharmacon	M-010946-02
RTN4 (siRNA pool)	Dharmacon	M-010721-00
RTN1 (siRNA pool)	Dharmacon	M-014138-00
ATL2 (siRNA pool)	Dharmacon	M-014047-00
ATL3 (siRNA pool)	Dharmacon	M-010656-00
SREBP1 (siRNA pool)	Dharmacon	M-006891-01
CKAP4 (siRNA pool)	Dharmacon	L-012755-01
RRBP1 (siRNA pool)	Dharmacon	L-011891-02
ATF4 (siRNA pool)	Dharmacon	L-005125-00
REEP4 (siRNA pool)	Dharmacon	L-016343-02
REEP5 (siRNA pool)	Dharmacon	L-019467-01
REEP6 (siRNA pool)	Dharmacon	L-015555-02
CAMSAP2 (siRNA pool)	Dharmacon	L-022091-00
Human XBP1 RT-PCR (fwd)	IDT	GAATGAAGTGAGGCCAGTGG
Human XBP1 RT-PCR (rev)	IDT	ACTGGGTCCTTCTGGGTAGA
Human PERK qRT-PCR (fwd)	IDT	GTCCCAAGGCTTTGGAATCTGTC
Human PERK qRT-PCR (rev)	IDT	CCTACCAAGACAGGAGTTCTGG
Human CKAP4 qRT-PCR (fwd)	IDT	TTTCTCGGGCTGGTGCGTCCA
Human CKAP4 qRT-PCR (fwd)	IDT	GGACTCAAAAGTTCCAAATGTGGC
Human RRBP1 qRT-PCR (fwd)	IDT	TCCTGTCTGAGAAGGCTGGCAT
Human RRBP1 qRT-PCR (rev)	IDT	CCTCAGTTTGCTCTTGGCGACA
Human REEP4 (qRT-PCR) fwd	IDT	GAGTGTTGGTCAGATACTGAGGC
Human REEP4 (qRT-PCR) rev	IDT	TTTCCTCGTCCGAACCTTCAGG
Human CAMSAP2 (qRT-PCR) fwd	IDT	GGAGGTCAAAAGGCTCGTTATCG
Human CAMSAP2 (qRT-PCR) rev	IDT	GGCAGCTAATGCACAGCCATCT
Human PERK esiRNA preparation	Sigma	Cat. Num. EHU030881
Human CAMSAP2 esiRNA preparation	Sigma	Cat. Num. EHU134911
Human RRBP1 esiRNA preparation	Sigma	Cat. Num. EHU073471

**Recombinant DNA**		

AcGFP-Sec61β	Addgene	Cat. Num. #15108; RRID:Addgene_15108
pRRL-GFP-Sec61 β -IRES-mCherry	This study	N/A
pRRL-IRES-EGFP	CNIC core services repository	N/A
eIF2α wild type	Addgene	Cat. Num. #21807; RRID:Addgene_21807
eIF2 α Ser51D	Addgene	Cat.Num. #21809; RRID:Addgene_21809
pcDNA3.1- eIF2 α wild type	This study	N/A
pcDNA3.1- eIF2 α Ser51D	This study	N/A
pCMVR8.74	Addgene	Cat. Num. #22036; RRID:Addgene_22036
pMD2.G	Addgene	Cat. Num. #12259; RRID:Addgene_12259
full-length p180 (lentiviral vector)	This study	N/A
p180MT deletion mutant RRBP1ΔCC (lentiviral vector)	This study	N/A

**Software and algorithms**		

Analysis pipeline script (Acapella software)	This study	
Sequest HT algorithm	Thermo Fisher Scientific	
Proteome Discoverer 2.5	Thermo Fisher Scientific	
iSanxot	Jesús Vázquez	Rodríguez et al.^71^
Generic Integration Algorithm	Jesús Vázquez	García-Marqués et al.^72^
DecoyPyrat		(https://www.longdom.org/open-access/decoypyrat-fast-nonredundant-hybrid-decoy-sequence-generation-forlarge-scale-proteomics-jpb-1000404.pdf)

**Other**		

384-well CellCarrier optical plates	PerkinElmer	Cat. Num. 6057308
96-well ViewPlate optical plates	PerkinElmer	Cat. Num. 78590-1

### Experimental model and study participant details

All cell maintenance and experiments were performed in a standard humidified incubator at 37°C and 5% CO_2_, unless otherwise stated. Low-passage MCF10A cells, MCF10A AT cells (stably expressing a constitutively active HRasV12 GTPase), and MCF10A-derived stable cell lines were cultured in DMEM-F12 Glutamax medium (Gibco) supplemented, unless otherwise stated, with 5% heat-inactivated horse serum (Gibco), 1 μg/mL bovine insulin, 1 μM hydrocortisone, 50U cholera toxin, and 100 ng/mL epidermal growth factor (EGF)- selection (puromycin, 5 ng/mL) was applied in stable cell lines. HeLa, Sh-Sy5y and MDA-MB231 cells were grown in high glucose DMEM supplemented with 10% heat inactivated fetal bovine serum (Gibco). Media were further supplemented with 25 μM β-mercaptoethanol and 1X non-esential amino acid mixture (Gibco). MCF10A and MCF10A-ATI cells were a kind gift from Claire Isacke (ICR, UK); MCF10A/Fv2E-PERK^34^ and MCF10A/PERKΔC^35^ stable cell lines were generously provided by Julio Aguirre-Ghiso (Mount Sinai Hospital, USA). All cell lines were routinely checked for mycoplasma contamination using PCR procedures.

### Method details

#### Treatments, vital stainings and reagents

ER bulk content was analyzed by flow cytometry after brief pulse-labeling (5 min) with BODIPY FL-ER tracker (Molecular Probes, ThermoScientific) cells freshly resuspended cells after indicated treatments, and analyzed on an LSR Fortessa station (Becton Dickinson). Tunicamycin, MG-132, puromycin, sodium arsenite, cicloheximide, nocodazole, guanabenz and Hoescht 33258 were obtained from Sigma. Thapsigargin was obtained from Santa Cruz Biotechnology. The GSK2606414 PERK inhibitor and harringtonin were purchased from Tocris. AP20187 homodimerizer was purchased from Selleckchem, centrinone was purchased from R&D systems. Methanol-free, 16% paraformaldehyde-PBS was purchased from ThermoScientific. Secondary antibodies and fluorescent conjugates were purchased from Molecular Probes. siRNAs were obtained from Dharmacon, esiRNAs were purchased from Sigma. Primary antibodies and siRNA/esiRNAs used are available in Key Resources Table.

#### cDNA constructs, establishment of stable cell lines, and transient transfections

For siRNA reverse transfection, Lipofectamine RNAiMAX (LifeSciences) reagent was used following supplier’s recommendations. Plasmid transfections were performed using Lipofectamine 3000 (LifeSciences) for 24 h. MCF10A-EGFP-Sec61β cell line was obtained by lentiviral transduction (MOI: 5; Viral Vector unit, CNIC, Spain; lentiviral vector cloned on pRRL-IRES-mCherry from NdeI/MluI digested parent AcGFP-Sec61β vector Addgene #15108) and two subsequent rounds of stringent sorting (FACS Aria, BD Biosciences) of EGFP positive cells with a fluorescence range within one order. Reverse transfection proceedings for transient silencing have been detailed elsewhere. For neurite extension assays, SH-Sy5y cells lentivirally transduced with a pRRL-IRES-EGFP vector (MOI: 1; Viral Vector unit, CNIC, Spain) were reverse transfected with indicated esiRNAs and incubated for 48h, then switched to differentiation medium (1.5% FBS, 1 μg/mL bovine insulin, 1μM hydrocortisone, 10 μM *trans*-retinoic acid (Sigma), and 5U brain-derived neurotropic factor (BDNF, Sigma)) and incubated for a further 48 h before being processed for immunofluorescence. eIF2α WT and S51D constructs were cloned on pcDNA3.1-HA from Addgene constructs #21807 and #21809. In experiments detailed in Figure 4A, where simultaneous transfection of cDNA constructs was required, cells were reverse transfected as detailed above, and 24 h after directly transfected using the Lipofectamine 3000 reagent. The lentiviral constructs expressing full-length p180 or the p180MT deletion mutant (lacking the coiled-coil region; RRBP1ΔCC) are based on a previous seminal report^45^ and were synthesized by GenScript service. Cells shown in experiments 6I and S5H,I were transduced at MOI = 10 (CNIC Viral Vector service) for 48 h before analysis.

#### High-throughput assays, immunostaining and acquisition

For high content imaging, cells were reverse transfected using Lipofectamine RNAiMAX reagent, and 40 ng of siRNA, on optical CellCarrier 384-well plates, on a final volume of 40 μL. Liquid handling, fixation and immunostaining was performed using a robotic station (HCS Explorer, PerkinElmer) according to previously detailed protocols.^31^ Briefly, samples were fixed by adding 40 μL of 8% paraformaldehyde in PBS for 20 min, washed thrice in PBS, permeabilized in 0.2% Triton X-100 for 15 min and blocked in 2% BSA in PBS for 1 h. Primary antibody staining was conducted overnight on 10 μL of blocking solution; after 3 washes in PBS, secondary antibodies and Hoeschtt 33342 were incubated for 90 min. After 3 washes, samples were stored in free PBS containing 0.005% sodium azide and imaged within a maximum 15 days. Acquisition and automated image analysis was performed with an Opera HCS II spinning disk confocal microscope.

#### Superesolution confocal microscopy

A Leica SP8 3X stimulated emission depletion (STED) spectral confocal microscope, equipped with hybrid photomultipliers, a CCD digital camera and an STED station with two separate depletion lasers (592 and 660nm) was used, with a 100X/1.4NA immersion objective. Sequential (between stacks) acquisition used 100% power for STED depletion and 70% power for illumination. Samples were prepared according to the manufacturer’s recommendations.

#### Image analysis

Image analysis proceedings are detailed together with full script file (based on Acapella Studio suite; PerkinElmer; data file D1), and comprehended the following steps: (1) nuclei and cell segmentation; (2) filtering of artifacts and out-of-focus objects; (3) definition of subcytoplasmic regions, and (4) gathering of ER-related features. The ER expansion ratio feature is calculated by first defining a perinuclear and a peripheral region in a single focal plane image, by setting a fixed proportional distance lying between the cell external boundary and the nucleus boundary, as previously described.^31^ Average pixel intensities for each region are captured, and the ratio between the peripheral region and the perinuclear region is used as “expansion ratio”. ImageJ analysis pipelines used in experiments shown in Figures 6 and 7 have been previously reported.^43^ Briefly, cell protrusions were manually segmented and number and relative area to cell body area were computed with ImageJ. Polarity index was calculated as *Pi= ∑(1-|sinα*_*i*_*|)·L*_*i*_*/∑L*_*i*_, whereby *α*_*i*_ is the angle between each protrusion _*i*_ and the longest protrusion, and *L*_*i*_ is the length of the given protrusion. The relative length of largest protrusion shown in Figures 7G and S6B is estimated as the ratio between the length of the largest protrusion and the major axis of the cell body.

#### Protein analysis

Cell lysates were harvested in colorless, non-reducing sample buffer (2% SDS, 150 KCl, 20 mM Tris pH6 .8, 10% glycerol, protease and phosphatase inhibitors), quantitated through Bradford assay and normalized, and supplemented with DTT and bromophenol blue to a final concentration of 0.2 mM and 0.012%, respectively. ∼10 μg/sample were loaded on 10–12.5% SDS-polyacrylamide gels, with the exception of gels used to analyzed CAMSAP2 protein levels, where 7% gels were used. After electrophoretic separation and blotting in conventional non-SDS, 20% methanol conditions to PVDF membranes, samples were probed with the indicated antibodies according to standard protocols.^31^ Signal was developed with the ECL Plus system (PerkinElmer).

#### Quantitative proteomics

Cultured cells were lysed using a lysis buffer containing 50 mM Tris-HCl (pH 7.8), 2% SDS, and 5 mM Tris-(2-Carboxyethyl)phosphine (TCEP). The samples were boiled for 5 min and then incubated at room temperature for 20 min to solubilize the proteins. After centrifugation at 15,000g for 15 min, the supernatants were collected, and protein concentration was measured using the Direct Detect system (Millipore).

Protein extracts were digested using the FASP method with 30 kDa centrifugal filter devices (NanoSep 30k Omega, Pall Life Sciences). Proteins were digested overnight at 37°C with modified trypsin (Promega) at a 40:1 protein-to-trypsin (w/w) ratio, and the resulting peptides were desalted using Oasis-HLB columns (Waters).

For stable isobaric labeling, the tryptic peptides were dissolved in 150 mM Triethylammonium bicarbonate (TEAB) buffer, and peptide concentration was determined using the Direct Detect system (Millipore). Equal amounts of each peptide sample were labeled with TMT 11plex reagents (Thermo Fisher) according to the manufacturer’s protocol, with minor modifications. After a 1-h incubation at room temperature, the reaction was stopped with 0.5% TFA, incubated for 10 min, and the peptides were combined. Samples were concentrated using a Speed Vac, and excess labeling reagents were removed and desalted using Oasis-HLB columns.

An aliquot of this mixture was set aside for full proteome analysis. To increase proteome coverage, another aliquot was fractionated by high-pH reverse-phase chromatography using the High pH Reversed-Phase Peptide Fractionation Kit (Pierce) following the supplier’s protocol.

High-resolution analysis of TMT-labelled peptides was conducted using the Easy-NanoLC 1200 system (Thermo Scientific) coupled to a Tribrid Orbitrap Fusion mass spectrometer (Thermo Scientific). Peptides were suspended in Buffer A (0.1% formic acid), loaded onto a pre-column (PepMap100 C18 LC, 75 μm ID, 2 cm, Thermo Scientific), and separated online on a NanoViper PepMap100 C18 LC analytical column (75 μm ID, 50 cm, Thermo Scientific). The separation was achieved using a continuous gradient of 5–32% B over 240 min and 32–90% B over 5 min (B = 100% acetonitrile, 0.1% formic acid) at a flow rate of 200 nL/min. Each MS run included enhanced FT-resolution spectra (60,000 resolution) in the 400–1,500 m/z range, followed by data-dependent MS/MS spectra of the most intense parent ions acquired during the chromatographic run. Dynamic exclusion was set to 40 s.

For peptide identification, the MS/MS spectra were searched using the Sequest HT algorithm implemented in Proteome Discoverer 2.5 (Thermo Fisher Scientific) against the Uniprot database containing all human sequences (June 2022), concatenated with decoy sequences generated using DecoyPyrat (https://www.longdom.org/open-access/decoypyrat-fast-nonredundant-hybrid-decoy-sequence-generation-forlarge-scale-proteomics-jpb-1000404.pdf). The parameters were set as follows: trypsin digestion with a maximum of 2 missed cleavages, precursor and fragment mass tolerances of 2 Da and 0.03 Da, respectively, carbamidomethyl (+57.021) cysteine and TMT modifications (+229.163) at peptide N-terminal and Lys residues as fixed modifications, and methionine oxidation (+15.995), proline hydroxylation (+15.995), phosphorylation of Ser, Thr, and Tyr residues (+79.966), and protein N terminus acetylation (+42.011) as dynamic modifications. The mass spectrometry proteomics data have been deposited to the ProteomeXchange Consortium via the PRIDE partner repository with the dataset identifier PXD057012.

For quantitative analysis, the iSanXoT program^71^ was utilized. False discovery rate (FDR) was calculated using the corrected Xcorr score (cXcorr)^73^ —which relies on the expectation maximization algorithm to run an analysis that instructs on learns correct vs. incorrect database search results, computing probabilities that peptide assignments to spectra are correct based upon database search scores and the number of tryptic termini of peptides. —with additional filtering for a precursor mass tolerance of 10 ppm. Identified peptides had an FDR of 1% or lower. Quantitative data from TMT reporter intensities were integrated from the spectrum level to the peptide level and then to the protein level to quantify the relative abundance of each protein using the WSPP (weighted spectrum, peptide, and protein) statistical model^74^ and the Generic Integration Algorithm (GIA).^72^ Briefly, these models standardize peptide and protein abundance quantification as log2-ratios, with values expressed in units of standard deviation for proteins (Zq) based on their estimated variances. Differences in peptide and protein abundance or functional behavior were estimated by comparing the groups’ Zq or Zc medians, respectively, as determined by the WSPP statistical model. Heatmaps are represented through the MORPHEUS open-source software (https://software.broadinstitute.org/morpheus/).

#### Puromycilation assay

Our protocol was modified from procedures published previously.^37^ Briefly, cells were exposed in a ∼45″ pulse to a low concentration of puromycin (500 ng/mL) and immediately washed twice in complete medium and fixed with 4% PFA. Samples were permeabilized 15 min in 0.2% Triton X-100 PBS, washed twice in 0.05% TX-100 PBS, and blocked for 2 h with 2% BSA in PBS. Standard immunofluorescence procedures were thereon applied to label with the 2B4 anti-puromycin monoclonal antibody, developed by A. David and coworkers^37^ and publicly available through the Developmental Studies Hybridoma Bank repository (entry ♯PMY-24B)

#### Choline incorporation assay

All reagents used were from Jena Bioscience (Jena, Germany). Cells undergoing siRNA-mediated knockdown and/or ER stress pharmacological induction as indicated, were exposed to 100 μM propargylcholine (ref. CLK-066) for 3 h before 4% PFA fixation and processing for immunofluorescence as described above. Samples were then labeled with CLICK chemistry reactions (ref. CLK-074) bearing AlexaFluor-647-azide derivative (ref. CLK-1299) as indicated by the supplier, imaged by spinning confocal microscopy and quantitated using the Acapella Studio software (PerkinElmer), using α-tubulin counterstaining for normalization. Median values of 10 independent biological replicates were used.

#### Proximity ligation assay

The DuoLink Orange commercial kit (Sigma-Aldrich) was used on image-bottom multiwell plates (PerkinElmer) where samples had been fixed, permeabilized, blocked and stained with a rabbit antiRRBP1 antibody (Proteintech) and a mouse anti-αtubulin (Sigma), at 1:2000 and 1:5000 concentrations according to procedures describe above (see STAR key resource table for details). PLA reactions were conducted and mounted as recommended by the supplier, and image acquired on an Opera microscope. Using the Columbus image analysis suite, regions corresponding to cell bodies were segmented from DAPI nuclear signal and cytoplasmic background, and dot-like bright structures segemented using the Spot analysis function. This signal above background was integrated for each cell, and well average values (40 fields, approx. 1200 cells) were computed.

#### Cell viability assays

Cell viability was inferred by the MTT  colorimetric assay. Briefly, cells were treated with either vehicle (DMSO) or tunicamycin (5 μg/mL, 36 h), and then exposed to 50 μg/mL MTT (3-(4,5-dimethylthiazol-2-yl)-2,5-diphenyltetrazolium bromide at 37°C for 3 h in the dark. Precipitated formazan was solubilized in DMSO, and absorption was measured at 542 nm in a spectrophotometer. Absorption values were referred to control condition as 100%.

#### Transwell migration assays

8.0 μm Ø-pore PET hanging inserts were pre-coated with collagen as recommended by the supplier (Millipore). Cells were seeded in a 500 μL volume of reverse transfection mixture and allowed to migrate for ∼4 h. Inserts were then placed on pre-warmed 4% PFA-PBS, and processed for immunostaining following standard protocols. Images were acquired on a Zeis Axiovert confocal microscope. Basal (trans) fluorescence was related to top (cis) fluorescence from the actin channel as segmented and quantitated by ImageJ standard tools from raw.lif images.

#### Culture on collagen matrices

∼100 μL Fibrillar dermal bovine collagen I layers (1.7 mg/mL; Corning) were casted on 96-well optical ViewPlate plates (PerkinElmer) following manufacturer's recommendations. ∼4000 (MCF10A, MCF10-ATI) or ∼6500 (MDA-MB231) cells were reverse transfected and plated on top of the pre-casted collagen plugs, on a total volume of 50μL of complete medium. After 48 h, cells were pulse-labelled with CellTracker Orange 561 (Molecular Probes), fixed by adding 50 μL of pre-warmed 16% PFA in PBS, and counterstained by adding 10 ng of Hoeschtt 33342. Acquisition of focal stacks was performed on an Opera HCS II spinning disk microscope, and in-focus images were manually selected for further analysis.

#### 3D collagen invasion assays

96-well optical glass plates (PerkinElmer) were coated overnight at 37°C with a sterile 1% solution of BSA to minimize cell attachment. Cells reverse transfected for 24 h were detached and embedded in a 1.7 mg/mL fibrillar dermal bovine collagen I matrix (Corning) at a density of 50000 cells/mL, and 150 μL were plated per well. Plates were immediately spun at 300 g for 5min and incubated for ∼4 h at 37°C to allow for collagen polymerization. 50 μL completion media (4X growth medium) containing the indicated treatments was added, and cells were allowed to invade the matrix overnight. Subsequently, samples were fixed and counterstained adding 50 μL of 16% PFA in PBS containing 500 ng/mL of Hoeschtt 33342. Plates were then imaged using an Operetta HCS system (PerkinElmer; ICR, London, UK), acquiring three optical planes with a spacing of ∼50μm. Nuclei segmentation and counting per plane was performed using the Columbus automated system (PerkinElmer).

### Quantification and statistical analysis

Statistical significance analyses and description of quantitative panels across figures are specified in each corresponding figure legend. Briefly, statistical significance analyses using Student’s t test were performed using the GraphPad Prism 10 suite; significance values are indicated as ^∗^: *p* < 0.05; ^∗∗^: *p* < 0.01; ^∗∗∗^: *p* < 0.005. n.s.: *p* > 0.05. All graphs represent either mean values with standard deviation (bar graphs) or individual replicates with their mean value (dot plots); boxplots show individual data ranges (error bars) and average values (middle box line). Dot plots in panels in Figures 3B, 3C, and S3C depict single-cell values; lines indicate regression analyses for correlation.

## Supplemental information

Document S1. Figures S1–S6 and Table S1

Data S1. Automated image analysis script used in high-content screening procedures, related to Figures 1 and S1 and Table S1

Document S2. Article plus supplemental information
