# Supplementary material for: PERK-dependent reciprocal crosstalk between ER and non-centrosomal microtubules coordinates ER architecture and cell shape
Source: Cell Rep. 2025 Apr 22;44(5):115590. doi: 10.1016/j.celrep.2025.115590 (PMC12548782; doi:10.1016/j.celrep.2025.115590)
Supplement: Document S1. Figures S1–S6 and Table S1 [file mmc1.pdf]

Cell Reports, Volume *44*

Supplemental information

PERK-dependent reciprocal crosstalk between ER and non-centrosomal microtubules coordinates ER architecture and cell shape

Miguel Sánchez-Álvarez, Fidel Nicolás Lolo, Heba Sailem, Giulio Fulgoni, Patricia Pascual-Vargas, Lucía Agüera, Mauro Catalá-Montoro, Mar Arias-García, Juan Antonio López, Jesús Vázquez, Miguel Ángel del Pozo, and Chris Bakal

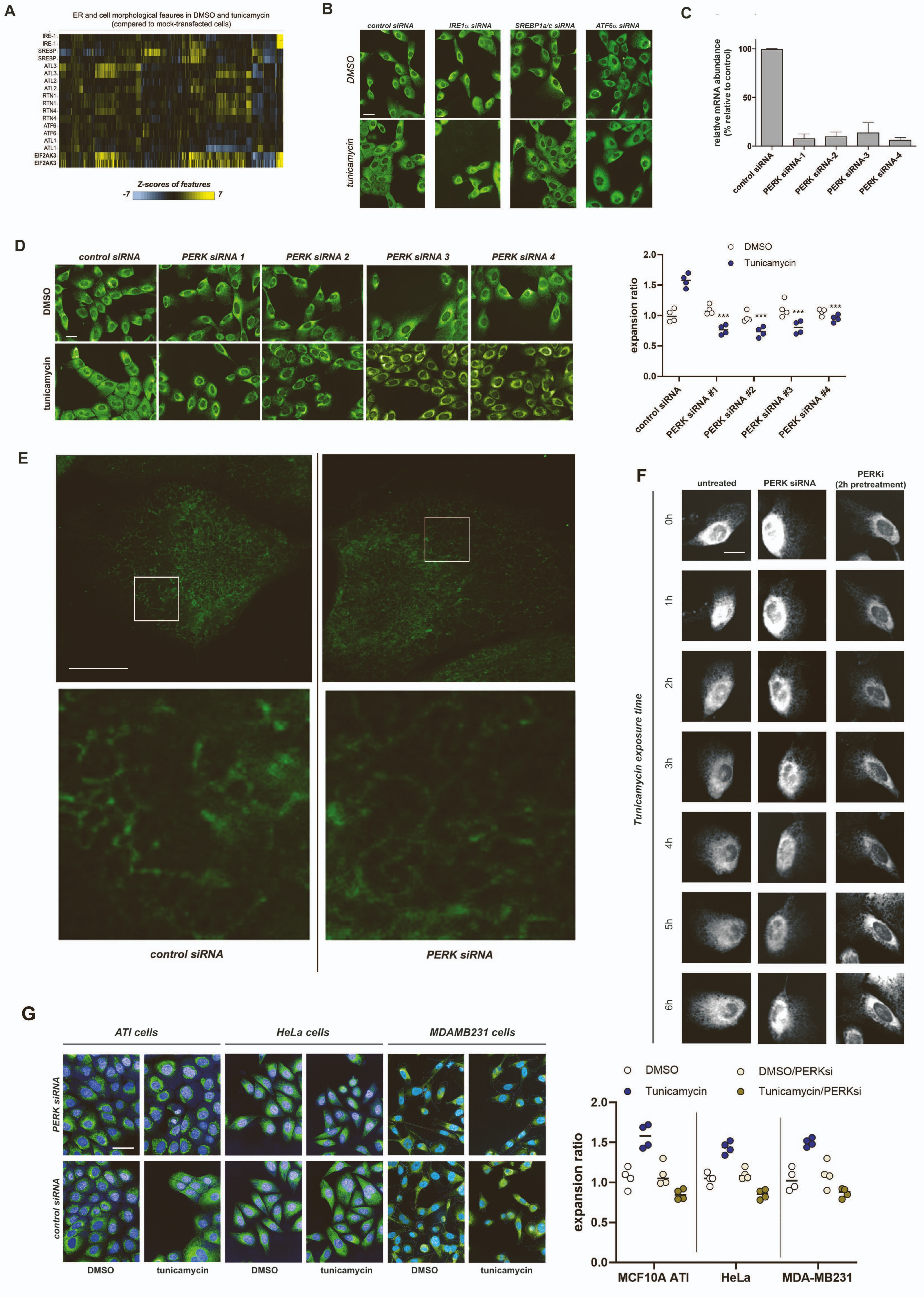

A
ER and cell morphological feaures in DMSO and tunicamycin
(compared to mock-transfected cells)
IRE-1
IRE-1
SREBP
SREBP
ATL3
ATL3
ATL2
ATL2
RTN1
RTN1
RTN4
RTN4
ATF6
ATF6
ATL1
ATL1
EIF2AK3
EIF2AK3
Z-scores of features
-7
7
B
control siRNA
IRE1α siRNA
SREBP1a/c siRNA
ATF6α siRNA
DMSO
tunicamycin
C
relative mRNA abundance
(% relative to control)
100
50
0
control siRNA
PERK siRNA-1
PERK siRNA-2
PERK siRNA-3
PERK siRNA-4
D
control siRNA
PERK siRNA 1
PERK siRNA 2
PERK siRNA 3
PERK siRNA 4
DMSO
tunicamycin
DMSO
Tunicamycin
expansion ratio
2.0
1.5
1.0
0.5
0.0
***
control siRNA
PERK siRNA #1
PERK siRNA #2
PERK siRNA #3
PERK siRNA #4
E
control siRNA
PERK siRNA
F
untreated
PERK siRNA
PERKi
(2h pretreatment)
Tunicamycin exposure time
0h
1h
2h
3h
4h
5h
6h
G
ATI cells
HeLa cells
MDAMB231 cells
PERK siRNA
control siRNA
DMSO
tunicamycin
DMSO
tunicamycin
DMSO
tunicamycin
DMSO
Tunicamycin
DMSO/PERKsi
Tunicamycin/PERKsi
expansion ratio
2.0
1.5
1.0
0.5
0.0
MCF10A ATI
HeLa
MDA-MB231

Supplementary Figure 1.- An image-based automated screening pipeline to explore the genetic control of ER architecture identifies PERK/EIF2AK3 as required for ER stress-induced ER remodeling (A) Heatmap showcasing Z-scores extracted from 2 biological replicates (averaged for 4 replicate wells for each condition) of all 136 image features (both including treated and non-treated conditions) across indicated siRNA pairs. Hierarchical clustering renders correct pairing of siRNA duplex replicas. See also Supplementary Table 1 for a complete list of image features. **(B)** Immunofluorescence images (calreticulin) of cells transfected with indicated siRNA duplexes and exposed to either vehicle (DMSO) or 1μg/ml tunicamycin for 6h. Note impaired remodeling in IRE1 and SREBP1a/c-depleted cells. Scale bar indicates 15microns. **(C, D)** Validation with independent siRNA sequences. [C] RT-PCR analysis of *EIF2AK3* mRNA levels in cells transfected with indicated siRNA duplexes. Data is derived from 3 technical replicates. [D] Representative unprocessed, automatically acquired images of anti-calreticulin-immunostained MCF10A cells across indicated conditions. Graphs derived from four independent replicates (~2000 cells per well). Scale bar indicates 15microns **(E) (related to figure 1E)** Superresolution images of cells solely exposed to DMSO vehicle. Scale bar indicates 10microns. **(F)** Live cell imaging of MCF10A cells stably expressing an EGFP-Sec61β fusion, across indicated times and treatments. Scale bar represents 10microns **(G)** Indicated cell lines were reverse transfected with an siRNA pool targeting PERK or scrambled, subjected to indicated treatments and processed for immunofuorescent staining (calreticulin; counterstained for DAPI), imaged and analysed. Graphs are derived from four independent replicates (~2000 cells per well). Scale bars indicate 15microns.

Statistical significance values from t-Student´s tests are indicated as *: $p < 0.05$; **: $p < 0.01$; ***: $p < 0.005$. n.s.: $p > 0.05$. Bar graph items show mean values (bar graphs) and standard deviation (error bars); dot plots represent individual replica values with their average indicated with an horizontal bar.

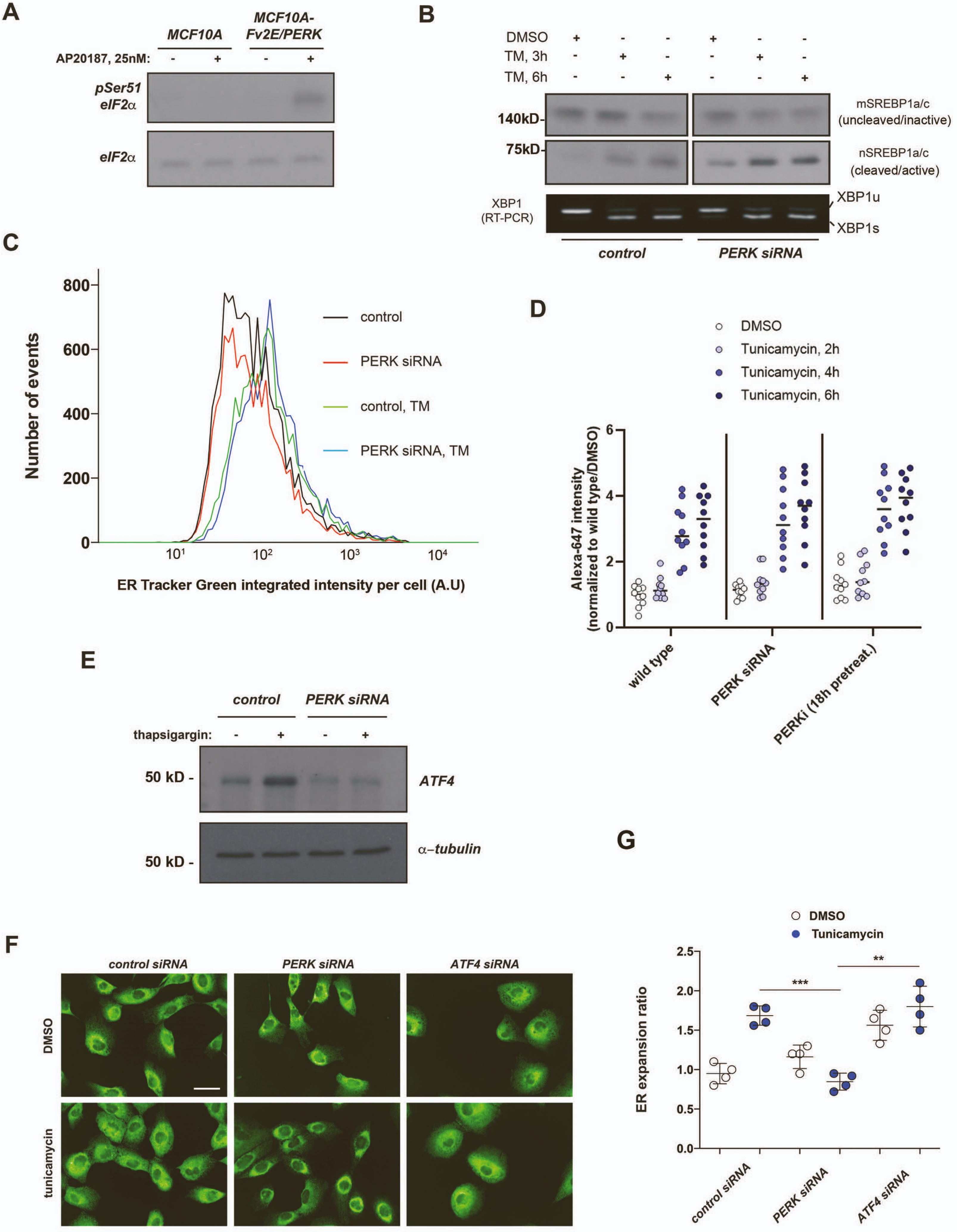
A
MCF10A
MCF10A-Fv2E/PERK
AP20187, 25nM: - + - +
pSer51 eIF2α
eIF2α
B
DMSO + - - + - -
TM, 3h - + - - + -
TM, 6h - - + - - +
140kD-
75kD-
mSREBP1a/c (uncleaved/inactive)
nSREBP1a/c (cleaved/active)
XBP1 (RT-PCR)
XBP1u
XBP1s
control
PERK siRNA
C
Number of events
800
600
400
200
0
10^1
10^2
10^3
10^4
control
PERK siRNA
control, TM
PERK siRNA, TM
ER Tracker Green integrated intensity per cell (A.U)
D
DMSO
Tunicamycin, 2h
Tunicamycin, 4h
Tunicamycin, 6h
Alexa-647 intensity (normalized to wild type/DMSO)
6
4
2
0
wild type
PERK siRNA
PERKi (18h pretreat.)
E
control
PERK siRNA
thapsigargin: - + - +
50 kD -
ATF4
50 kD -
α–tubulin
F
control siRNA
PERK siRNA
ATF4 siRNA
DMSO
tunicamycin
G
DMSO
Tunicamycin
ER expansion ratio
2.5
2.0
1.5
1.0
0.5
0.0
***
**
control siRNA
PERK siRNA
ATF4 siRNA

Supplementary Figure 2.- PERK-dependent ER architectural remodeling is distinct from de novo ER membrane synthesis and formation, and does not depend on ATF4/CHOP axis. (A) Whole-cell extracts from MCF10A or MCF10A-Fv2E-PERK cells treated as indicated, were analyzed by western blotting with indicated primary antibodies. **(B)** Protein and total RNA was extracted from cells treated as indicated, and analyzed by western blot and RT-PCR. **(C)** MCF10A cells treated as indicated were detached and labeled with ER Tracker-BODIPY FL for 15min, and analyzed by flow cytometry for ER total content. **(D)** Propargylcholine incorporation assay on MCF10A cells treated as indicated. For the last 3h of the experiments, cells were supplemented with propargylcholine, processed for immunofluorescence and labelled through CLIC chemistry with azide-Alexa647. α-tubulin-normalized Alexa 647 intensity was recorded for 10 independent biological replicates. All values are normalized to the control, untreated average. **(E-G)** MCF10A cells were reverse transfected with an siRNA pool targeting ATF4 or scrambled, subjected to indicated treatments and processed for immunofuorescent staining (calreticulin; counterstained for DAPI), imaged and analysed. Graphs are derived from four independent replicates (~2000 cells per well). Scale bars indicate 15microns.

Statistical significance values from t-Student´s tests are indicated as *: p <0.05; **: p <0.01; ***: p <0.005. n.s.: p >0.05. Bar graph items show mean values (bar graphs) and standard deviation (error bars); dot plots represent individual replica values with their average indicated with an horizontal bar.

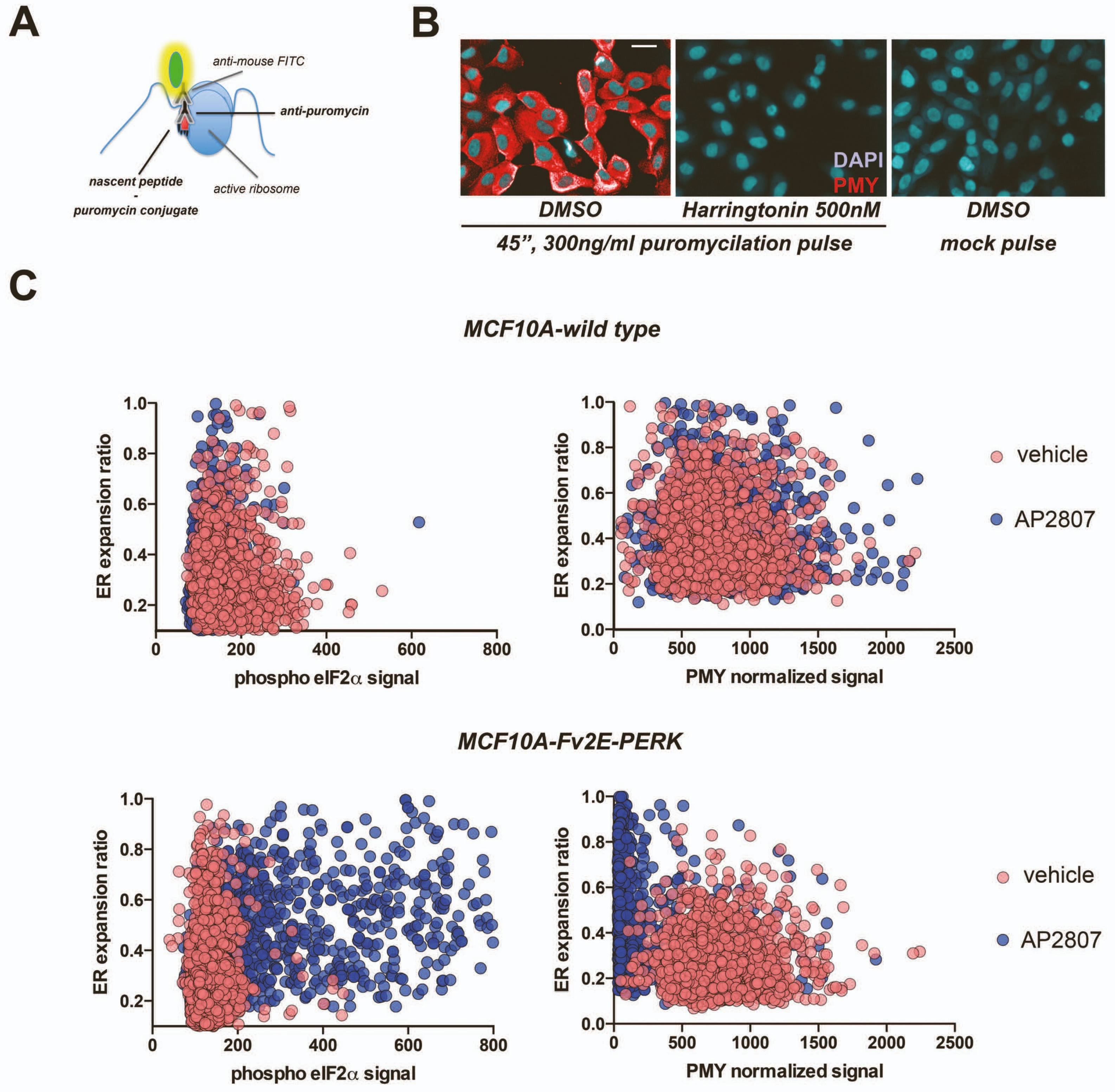
A
anti-mouse FITC
anti-puromycin
nascent peptide
-
puromycin conjugate
active ribosome
B
DAPI
PMY
DMSO
Harringtonin 500nM
DMSO
45", 300ng/ml puromycilation pulse
mock pulse
C
MCF10A-wild type
ER expansion ratio
phospho eIF2α signal
PMY normalized signal
vehicle
AP2807
MCF10A-Fv2E-PERK
ER expansion ratio
phospho eIF2α signal
PMY normalized signal
vehicle
AP2807

Supplementary Figure 3.- *In situ* puromycylation allows for single cell-level correlation of ER architecture and protein translation activity. (A) Diagram of puromycylation immunostaining technique **(B)** Control assay showing specificity of the technique. Scale bar indicates 15microns. **(C)** The synthetic Fv2E-PERK homodimerization system bypassing ER stress activation recapitulates the observations on ER expansion correlation and translation shutdown at single-cell level (related to figure 3B and C). Dot plots represent individual cell values.

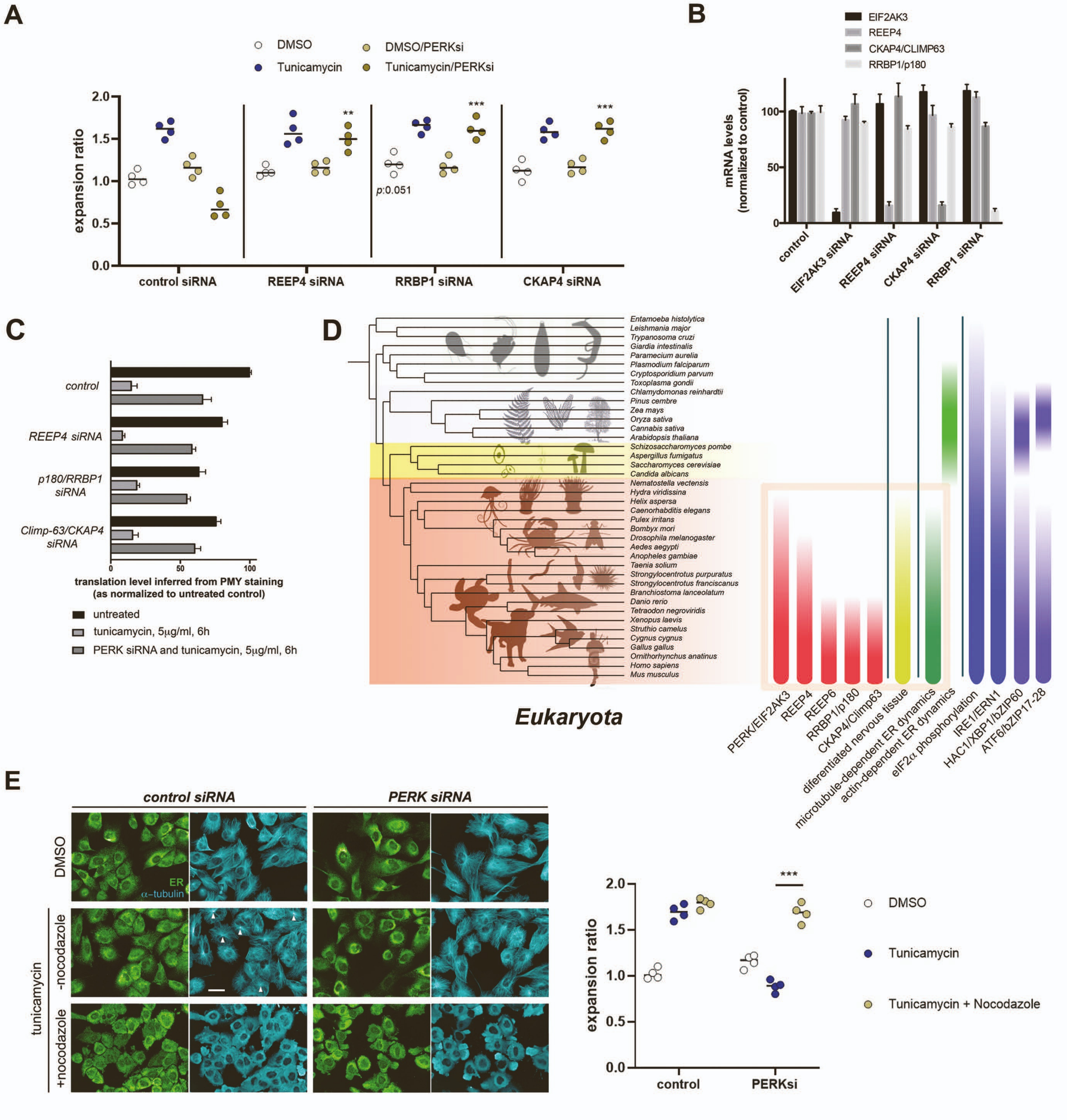

A
DMSO
Tunicamycin
DMSO/PERKsi
Tunicamycin/PERKsi
expansion ratio
p:0.051
control siRNA
REEP4 siRNA
RRBP1 siRNA
CKAP4 siRNA
B
EIF2AK3
REEP4
CKAP4/CLIMP63
RRBP1/p180
mRNA levels (normalized to control)
control
EIF2AK3 siRNA
REEP4 siRNA
CKAP4 siRNA
RRBP1 siRNA
C
control
REEP4 siRNA
p180/RRBP1 siRNA
Climp-63/CKAP4 siRNA
translation level inferred from PMY staining (as normalized to untreated control)
untreated
tunicamycin, 5μg/ml, 6h
PERK siRNA and tunicamycin, 5μg/ml, 6h
D
Entamoeba histolytica
Leishmania major
Trypanosoma cruzi
Giardia intestinalis
Paramecium aurelia
Plasmodium falciparum
Cryptosporidium parvum
Toxoplasma gondii
Chlamydomonas reinhardtii
Pinus cembre
Zea mays
Oryza sativa
Cannabis sativa
Arabidopsis thaliana
Schizosaccharomyces pombe
Aspergillus fumigatus
Saccharomyces cerevisiae
Candida albicans
Nematostella vectensis
Hydra viridissina
Helix aspersa
Caenorhabditis elegans
Pulex irritans
Bombyx mori
Drosophila melanogaster
Aedes aegypti
Anopheles gambiae
Taenia solium
Strongylocentrotus purpuratus
Strongylocentrotus franciscanus
Branchiostoma lanceolatum
Danio rerio
Tetraodon negroviridis
Xenopus laevis
Struthio camelus
Cygnus cygnus
Gallus gallus
Ornithorhynchus anatinus
Homo sapiens
Mus musculus
Eukaryota
PERK/EIF2AK3
REEP4
REEP6
RRBP1/p180
CKAP4/Climp63
differentiated nervous tissue
microtubule-dependent ER dynamics
actin-dependent ER dynamics
eIF2α phosphorylation
IRE1/ERN1
HAC1/XBP1/bZIP60
ATF6/bZIP17-28
E
control siRNA
PERK siRNA
DMSO
ER
α-tubulin
tunicamycin
-nocodazole
+nocodazole
expansion ratio
control
PERKsi
DMSO
Tunicamycin
Tunicamycin + Nocodazole

Supplementary Figure 4.- ER-microtubule tethers modulate ER stress-induced ER architectural remodeling. (A) ER expansion values across indicated conditions from the focused screen shown in figure 5 (4 biological replicates, approx. 2000 cells each). **(B)** qRT-PCR analysis of indicated mRNA transcripts across indicated conditions. Data are derived from 3 biological replicates. **(C)** Image-based puromycylation assay across indicated conditions (4 biological replicates, approx. 2000 cells each). Data are expressed as normalized to control untreated condition, which is set as 100%. **(D)** Visual rendering of estimated evolutionary conservation of indicated genes, as related to specific features of ER-cytoskeleton relationship. Evolutionary conservation is derived from InParanoid database, and layered over an evolutionary tree across indicated eukaryotic phila (OrthoDB, University of Geneva). **(E)** MCF10A cells were subjected to indicated siRNA and small compound treatments (tunicamycin 5μg/ml, nocodazole 20nM; 6h), fixed and immunostained for ER and microtubules, and imaged. Arrowheads indicate cells with clear radial disposition of microtubules.

Statistical significance values from t-Student´s tests are indicated as *: $p < 0.05$; **: $p < 0.01$; ***: $p < 0.005$. n.s.: $p > 0.05$. Bar graph items show mean values (bar graphs) and standard deviation (error bars); dot plots represent individual values with their average indicated with an horizontal bar.

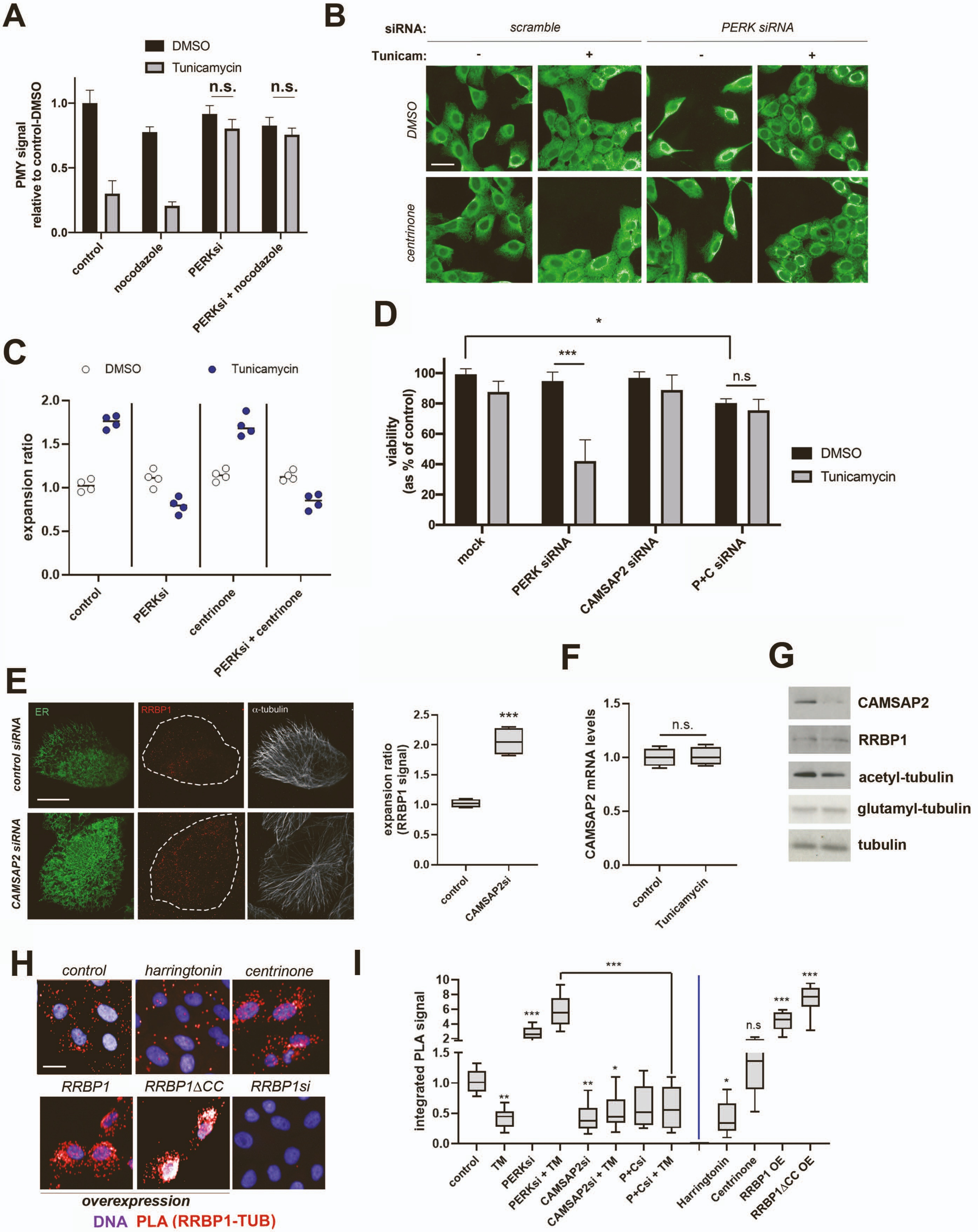

A
DMSO
Tunicamycin
PMY signal relative to control-DMSO
n.s.
n.s.
control
nocodazole
PERKsi
PERKsi + nocodazole
B
siRNA:
scramble
PERK siRNA
Tunicam:
DMSO
centrinone
C
DMSO
Tunicamycin
expansion ratio
control
PERKsi
centrinone
PERKsi + centrinone
D
viability (as % of control)
***
n.s
DMSO
Tunicamycin
mock
PERK siRNA
CAMSAP2 siRNA
P+C siRNA
E
ER
RRBP1
α-tubulin
control siRNA
CAMSAP2 siRNA
expansion ratio (RRBP1 signal)
control
CAMSAP2si
F
CAMSAP2 mRNA levels
n.s.
control
Tunicamycin
G
CAMSAP2
RRBP1
acetyl-tubulin
glutamyl-tubulin
tubulin
H
control
harringtonin
centrinone
RRBP1
RRBP1ΔCC
RRBP1si
overexpression
DNA PLA (RRBP1-TUB)
I
integrated PLA signal
n.s
control
TM
PERKsi
PERKsi + TM
CAMSAP2si
CAMSAP2si + TM
P+Csi
P+Csi + TM
Harringtonin
Centrinone
RRBP1 OE
RRBP1ΔCC OE

Supplementary Figure 5.- A non-centrosomal microtubule population is specifically required for ER-MT tethering and ER architecture control. (A) Image-based puromycylation assay across indicated conditions (4 biological replicates, approx. 2000 cells each). Data are expressed as normalized to control untreated condition. **(B-C)** ER remodeling assay (immunofluorescence images: calreticulin) across indicated conditions (4 biological replicates, approx. 2000 cells each), in cells passaged thrice in the presence of 10nM centrinone. Scale bar: 15micron. **(D)** MTT viability assay across indicated conditions. Data were derived from eight biological replicates. **(E)** Immunostaining for RRBP/p180 (*red*) and α-tubulin (*grayscale*) in MCF10A cells stably expressing Sec61β-EGFP, transfected with indicated siRNAs. Cell boundary is highlighted with dashed lines. Scale bar: 5microns. **(F)** qRT-PCR for *CAMSAP2* mRNA from total RNA extracted from MCF10A cells either exposed to DMSO or tunicamycin, 5μg/ml 6h. Data is derived from 3 independent replicates. **(G)** Western blot analysis of whole cell lysates harvested from MCF10A cells transfected with indicated siRNAs, for indicated markers. **(H)** Spinning disk confocal images of proximity ligation assay samples assayed for RRBP1-αtubulin interaction [red signal] across indicated conditions. Harringtonin was applied for 60min as in Fig. 2E; centrinone treatment at 10nM was applied for three passages. Sacle bar: 10microns **(I)** Quantitation of PLA signal across experiments shown in Fig. 6G and S5H as detailed in Materials and Methods.

Significance values are indicated as *: $p < 0.05$; **: $p < 0.01$; ***: $p < 0.005$. n.s.: $p > 0.05$. All graphs represent either mean values with standard deviation (bar graphs) or individual replicates with their mean value (dot plots); box plots show individual data ranges (error bars) and average values (middle box line).

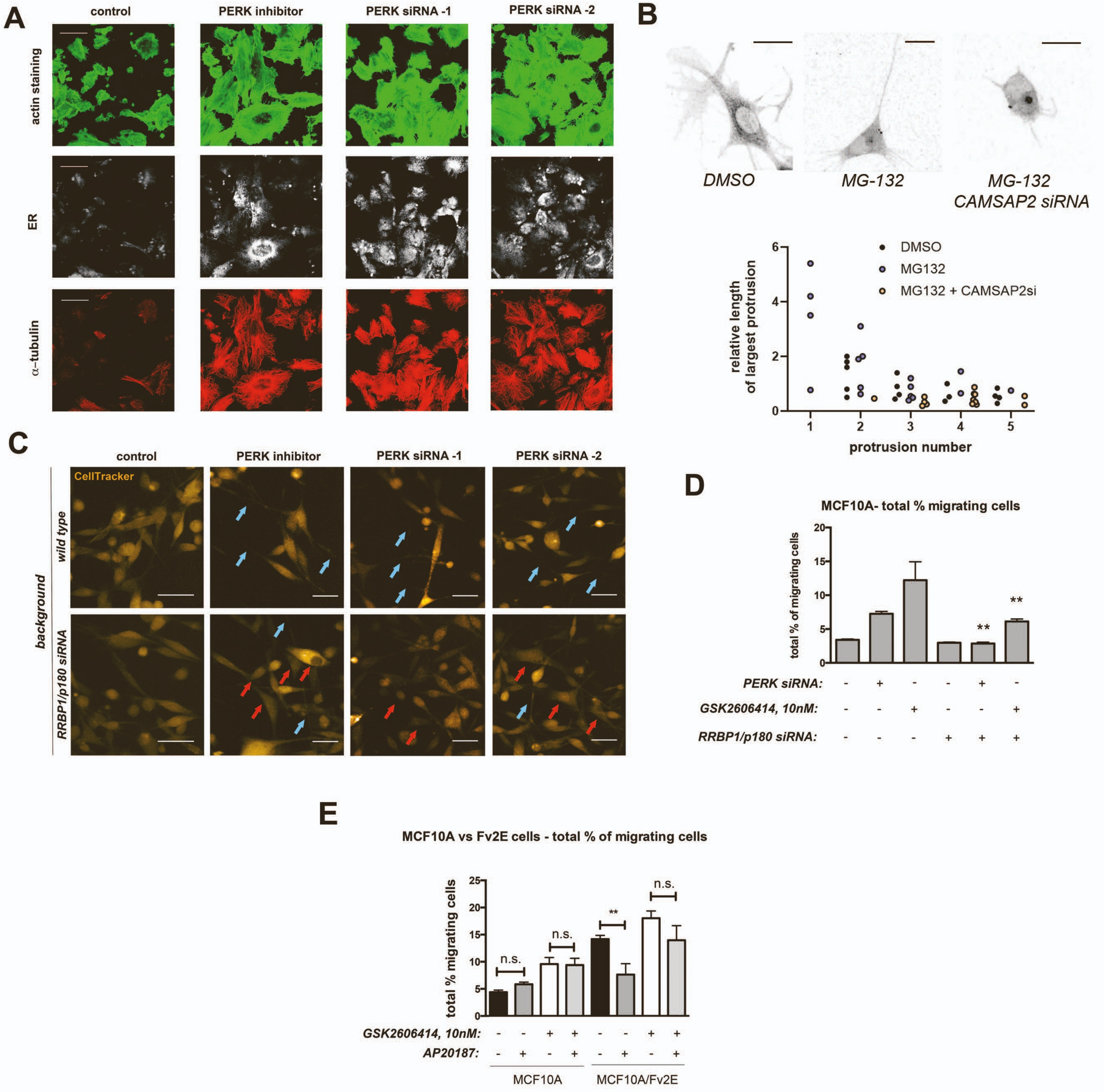

A
control
PERK inhibitor
PERK siRNA -1
PERK siRNA -2
actin staining
ER
α-tubulin
B
DMSO
MG-132
MG-132 CAMSAP2 siRNA
DMSO
MG132
MG132 + CAMSAP2si
relative length of largest protrusion
protrusion number
C
control
PERK inhibitor
PERK siRNA -1
PERK siRNA -2
CellTracker
background
wild type
RRBP1/p180 siRNA
D
MCF10A- total % migrating cells
total % of migrating cells
PERK siRNA:
GSK2606414, 10nM:
RRBP1/p180 siRNA:
E
MCF10A vs Fv2E cells - total % of migrating cells
total % migrating cells
n.s.
**
GSK2606414, 10nM:
AP20187:
MCF10A
MCF10A/Fv2E

Supplementary Figure 6.- ER-non-centrosomal MT coupling regulates cell morphology, and polarized protrussiveness and behavior. (A) Control Transwell™ protrusion formation assay in MCF10A cells across indicated conditions and labels. PERK inhibitor: GSK2606414, 40nM. Scale bar indicates 5microns. **(B; related to Fig. 7F-G)** SH-Sy5y neuroblasts treated as indicated were stimulated for neural differentiation (see M&Ms). The number of protrusions per cell, and the length of the longest protrusion relative to the perimeter of the cell soma are plotted for each condition (n=20 from 2 biological replicates). MG-132 treatment was 10nM, 48h. Scale bars indicate 10microns across micrographs. **(C)** Cells subjected to indicated siRNA treatments were plated on soft collagen matrices, counterstained with CellTracker Orange, and imaged by confocal microscopy. Examples of cells with long protrusions (blue arrows), as opposed to cells with no apparent elongated protrusions (red arrows), are indicated. Scale bar indicates 20microns **(D, E)** Cell migration assays across indicated conditions and MCF10A clones, related to experiments shown in Fig. 7F. Data were derived from 6 biological replicates.

Significance values from t-Student´s tests are indicated as *: $p < 0.05$; **: $p < 0.01$; ***: $p < 0.005$. n.s.: $p > 0.05$. All graphs represent mean values (bars) with standard deviation (error lines).

Supplementary Table 1.- List of acquired cell structure features in the high-content siRNA screen for genetic regulators of ER architecture.

Nuclei Selected - Number of Objects
Nuclei Selected Selected - Number of Objects
Nuclei Selected Selected - Nucleus Area [åµmå_] - Mean per Well
Nuclei Selected Selected - Nucleus Roundness - Mean per Well
Nuclei Selected Selected - Nucleus Ratio Width to Length - Mean per Well
Nuclei Selected Selected - Cell Area [åµmå_] - Mean per Well
Nuclei Selected Selected - Cell Roundness - Mean per Well
Nuclei Selected Selected - ER Formula - Mean per Well
Nuclei Selected Selected - ER Formula - CV % per Well
Nuclei Selected Selected - outer ER Channel3 Haralick Correlation 1 px - Mean per Well
Nuclei Selected Selected - outer ER Channel3 Haralick Correlation 1 px - CV % per Well
Nuclei Selected Selected - outer ER Channel3 Haralick Contrast 1 px - Mean per Well
Nuclei Selected Selected - outer ER Channel3 Haralick Contrast 1 px - CV % per Well
Nuclei Selected Selected - outer ER Channel3 Haralick Sum Variance 1 px - Mean per Well
Nuclei Selected Selected - outer ER Channel3 Haralick Sum Variance 1 px - CV % per Well
Nuclei Selected Selected - outer ER Channel3 Haralick Homogeneity 1 px - Mean per Well

Nuclei Selected Selected - outer ER Channel3 Haralick Homogeneity 1 px - CV % per Well
Nuclei Selected Selected - inner ER Channel3 SER Spot 1.75 px - Mean per Well
Nuclei Selected Selected - inner ER Channel3 SER Hole 1.75 px - Mean per Well
Nuclei Selected Selected - inner ER Channel3 SER Edge 1.75 px - Mean per Well
Nuclei Selected Selected - inner ER Channel3 SER Ridge 1.75 px - Mean per Well

Nuclei Selected Selected - inner ER Channel3 SER Valley 1.75 px - Mean per Well
Nuclei Selected Selected - inner ER Channel3 SER Saddle 1.75 px - Mean per Well
Nuclei Selected Selected - outer ER Symmetry 02 - Mean per Well
Nuclei Selected Selected - outer ER Symmetry 03 - Mean per Well
Nuclei Selected Selected - outer ER Symmetry 04 - Mean per Well
Nuclei Selected Selected - outer ER Symmetry 05 - Mean per Well
Nuclei Selected Selected - outer ER Symmetry 12 - Mean per Well
Nuclei Selected Selected - outer ER Symmetry 13 - Mean per Well
Nuclei Selected Selected - outer ER Symmetry 14 - Mean per Well
Nuclei Selected Selected - outer ER Symmetry 15 - Mean per Well
Nuclei Selected Selected - outer ER Threshold Compactness 30% - Mean per Well
Nuclei Selected Selected - outer ER Threshold Compactness 40% - Mean per Well
Nuclei Selected Selected - outer ER Threshold Compactness 50% - Mean per Well
Nuclei Selected Selected - outer ER Threshold Compactness 60% - Mean per Well
Nuclei Selected Selected - outer ER Axial Small Length - Mean per Well
Nuclei Selected Selected - outer ER Axial Length Ratio - Mean per Well
Nuclei Selected Selected - outer ER Radial Mean - Mean per Well
Nuclei Selected Selected - outer ER Radial Relative Deviation - Mean per Well
Nuclei Selected Selected - outer ER Profile 1/2 - Mean per Well
Nuclei Selected Selected - outer ER Profile 2/2 - Mean per Well
Nuclei Selected Selected - outer ER Symmetry 02 SP-Filter - Mean per Well

Nuclei Selected Selected - outer ER Symmetry 03 SP-Filter - Mean per Well
Nuclei Selected Selected - outer ER Symmetry 04 SP-Filter - Mean per Well
Nuclei Selected Selected - outer ER Symmetry 05 SP-Filter - Mean per Well
Nuclei Selected Selected - outer ER Symmetry 12 SP-Filter - Mean per Well
Nuclei Selected Selected - outer ER Symmetry 13 SP-Filter - Mean per Well
Nuclei Selected Selected - outer ER Symmetry 14 SP-Filter - Mean per Well
Nuclei Selected Selected - outer ER Symmetry 15 SP-Filter - Mean per Well
Nuclei Selected Selected - outer ER Threshold Compactness 30% SP-Filter - Mean per Well
Nuclei Selected Selected - outer ER Threshold Compactness 40% SP-Filter - Mean per Well
Nuclei Selected Selected - outer ER Threshold Compactness 50% SP-Filter - Mean per Well
Nuclei Selected Selected - outer ER Threshold Compactness 60% SP-Filter - Mean per Well
Nuclei Selected Selected - outer ER Axial Small Length SP-Filter - Mean per Well
Nuclei Selected Selected - outer ER Axial Length Ratio SP-Filter - Mean per Well
Nuclei Selected Selected - outer ER Radial Mean SP-Filter - Mean per Well
Nuclei Selected Selected - outer ER Radial Relative Deviation SP-Filter - Mean per Well
Nuclei Selected Selected - outer ER Radial Mean Ratio SP-Filter - Mean per Well
Nuclei Selected Selected - outer ER Profile 1/2 SP-Filter - Mean per Well
Nuclei Selected Selected - outer ER Profile 2/2 SP-Filter - Mean per Well
Nuclei Selected Selected - outer ER Symmetry 02 SER-Ridge - Mean per Well
Nuclei Selected Selected - outer ER Symmetry 03 SER-Ridge - Mean per Well
Nuclei Selected Selected - outer ER Symmetry 04 SER-Ridge - Mean per Well
Nuclei Selected Selected - outer ER Symmetry 05 SER-Ridge - Mean per Well
Nuclei Selected Selected - outer ER Symmetry 12 SER-Ridge - Mean per Well
Nuclei Selected Selected - outer ER Symmetry 13 SER-Ridge - Mean per Well
Nuclei Selected Selected - outer ER Symmetry 14 SER-Ridge - Mean per Well
Nuclei Selected Selected - outer ER Symmetry 15 SER-Ridge - Mean per Well
Nuclei Selected Selected - outer ER Threshold Compactness 30% SER-Ridge - Mean per Well
Nuclei Selected Selected - outer ER Threshold Compactness 40% SER-Ridge - Mean per Well
Nuclei Selected Selected - outer ER Threshold Compactness 50% SER-Ridge - Mean per Well
Nuclei Selected Selected - outer ER Threshold Compactness 60% SER-Ridge - Mean per Well
Nuclei Selected Selected - outer ER Axial Small Length SER-Ridge - Mean per Well
Nuclei Selected Selected - outer ER Axial Length Ratio SER-Ridge - Mean per Well
Nuclei Selected Selected - outer ER Radial Mean SER-Ridge - Mean per Well
Nuclei Selected Selected - outer ER Radial Relative Deviation SER-Ridge - Mean per Well
Nuclei Selected Selected - outer ER Radial Mean Ratio SER-Ridge - Mean per Well
Nuclei Selected Selected - outer ER Profile 1/2 SER-Ridge - Mean per Well
Nuclei Selected Selected - outer ER Profile 2/2 SER-Ridge - Mean per Well
Nuclei Selected Selected - outer ER Symmetry 02 SER-Valley - Mean per Well
Nuclei Selected Selected - outer ER Symmetry 03 SER-Valley - Mean per Well
Nuclei Selected Selected - outer ER Symmetry 04 SER-Valley - Mean per Well
Nuclei Selected Selected - outer ER Symmetry 05 SER-Valley - Mean per Well
Nuclei Selected Selected - outer ER Symmetry 12 SER-Valley - Mean per Well
Nuclei Selected Selected - outer ER Symmetry 13 SER-Valley - Mean per Well
Nuclei Selected Selected - outer ER Symmetry 14 SER-Valley - Mean per Well
Nuclei Selected Selected - outer ER Symmetry 15 SER-Valley - Mean per Well
Nuclei Selected Selected - outer ER Threshold Compactness 30% SER-Valley - Mean per Well
Nuclei Selected Selected - outer ER Threshold Compactness 40% SER-Valley - Mean per Well

Nuclei Selected Selected - outer ER Threshold Compactness 50% SER-Valley - Mean per Well
Nuclei Selected Selected - outer ER Threshold Compactness 60% SER-Valley - Mean per Well
Nuclei Selected Selected - outer ER Axial Small Length SER-Valley - Mean per Well
Nuclei Selected Selected - outer ER Axial Length Ratio SER-Valley - Mean per Well
Nuclei Selected Selected - outer ER Radial Mean SER-Valley - Mean per Well
Nuclei Selected Selected - outer ER Radial Relative Deviation SER-Valley - Mean per Well
Nuclei Selected Selected - outer ER Radial Mean Ratio SER-Valley - Mean per Well
Nuclei Selected Selected - outer ER Profile 1/2 SER-Valley - Mean per Well
Nuclei Selected Selected - outer ER Profile 2/2 SER-Valley - Mean per Well
Nuclei Selected Selected - outer ER Symmetry 02 SER-Hole - Mean per Well
Nuclei Selected Selected - outer ER Symmetry 03 SER-Hole - Mean per Well
Nuclei Selected Selected - outer ER Symmetry 04 SER-Hole - Mean per Well
Nuclei Selected Selected - outer ER Symmetry 05 SER-Hole - Mean per Well
Nuclei Selected Selected - outer ER Symmetry 12 SER-Hole - Mean per Well
Nuclei Selected Selected - outer ER Symmetry 13 SER-Hole - Mean per Well
Nuclei Selected Selected - outer ER Symmetry 14 SER-Hole - Mean per Well
Nuclei Selected Selected - outer ER Symmetry 15 SER-Hole - Mean per Well
Nuclei Selected Selected - outer ER Threshold Compactness 30% SER-Hole - Mean per Well
Nuclei Selected Selected - outer ER Threshold Compactness 40% SER-Hole - Mean per Well
Nuclei Selected Selected - outer ER Threshold Compactness 50% SER-Hole - Mean per Well
Nuclei Selected Selected - outer ER Threshold Compactness 60% SER-Hole - Mean per Well
Nuclei Selected Selected - outer ER Axial Small Length SER-Hole - Mean per Well
Nuclei Selected Selected - outer ER Axial Length Ratio SER-Hole - Mean per Well
Nuclei Selected Selected - outer ER Radial Mean SER-Hole - Mean per Well
Nuclei Selected Selected - outer ER Radial Relative Deviation SER-Hole - Mean per Well
Nuclei Selected Selected - outer ER Radial Mean Ratio SER-Hole - Mean per Well
Nuclei Selected Selected - outer ER Profile 1/2 SER-Hole - Mean per Well
Nuclei Selected Selected - outer ER Profile 2/2 SER-Hole - Mean per Well
Nuclei Selected Selected - outer ER Symmetry 02 SER-Saddle - Mean per Well
Nuclei Selected Selected - outer ER Symmetry 03 SER-Saddle - Mean per Well
Nuclei Selected Selected - outer ER Symmetry 04 SER-Saddle - Mean per Well
Nuclei Selected Selected - outer ER Symmetry 05 SER-Saddle - Mean per Well
Nuclei Selected Selected - outer ER Symmetry 12 SER-Saddle - Mean per Well
Nuclei Selected Selected - outer ER Symmetry 13 SER-Saddle - Mean per Well
Nuclei Selected Selected - outer ER Symmetry 14 SER-Saddle - Mean per Well
Nuclei Selected Selected - outer ER Symmetry 15 SER-Saddle - Mean per Well
Nuclei Selected Selected - outer ER Threshold Compactness 30% SER-Saddle - Mean per Well
Nuclei Selected Selected - outer ER Threshold Compactness 40% SER-Saddle - Mean per Well
Nuclei Selected Selected - outer ER Threshold Compactness 50% SER-Saddle - Mean per Well
Nuclei Selected Selected - outer ER Threshold Compactness 60% SER-Saddle - Mean per Well
Nuclei Selected Selected - outer ER Axial Small Length SER-Saddle - Mean per Well
Nuclei Selected Selected - outer ER Axial Length Ratio SER-Saddle - Mean per Well
Nuclei Selected Selected - outer ER Radial Mean SER-Saddle - Mean per Well
Nuclei Selected Selected - outer ER Radial Relative Deviation SER-Saddle - Mean per Well
Nuclei Selected Selected - outer ER Radial Mean Ratio SER-Saddle - Mean per Well
Nuclei Selected Selected - outer ER Profile 1/2 SER-Saddle - Mean per Well

Nuclei Selected Selected - outer ER Profile 2/2 SER-Saddle - Mean per Well
Nuclei Selected Selected - outer ER Symmetry 02 SER-Edge - Mean per Well
Nuclei Selected Selected - outer ER Symmetry 03 SER-Edge - Mean per Well
Nuclei Selected Selected - outer ER Symmetry 04 SER-Edge - Mean per Well
Nuclei Selected Selected - outer ER Symmetry 05 SER-Edge - Mean per Well
Nuclei Selected Selected - outer ER Symmetry 12 SER-Edge - Mean per Well
Nuclei Selected Selected - outer ER Symmetry 13 SER-Edge - Mean per Well
Nuclei Selected Selected - outer ER Symmetry 14 SER-Edge - Mean per Well
Nuclei Selected Selected - outer ER Symmetry 15 SER-Edge - Mean per Well
Nuclei Selected Selected - outer ER Threshold Compactness 30% SER-Edge - Mean per Well
Nuclei Selected Selected - outer ER Threshold Compactness 40% SER-Edge - Mean per Well
Nuclei Selected Selected - outer ER Threshold Compactness 50% SER-Edge - Mean per Well
Nuclei Selected Selected - outer ER Threshold Compactness 60% SER-Edge - Mean per Well
Nuclei Selected Selected - outer ER Axial Small Length SER-Edge - Mean per Well
Nuclei Selected Selected - outer ER Axial Length Ratio SER-Edge - Mean per Well
Nuclei Selected Selected - outer ER Radial Mean SER-Edge - Mean per Well
Nuclei Selected Selected - outer ER Radial Relative Deviation SER-Edge - Mean per Well
Nuclei Selected Selected - outer ER Radial Mean Ratio SER-Edge - Mean per Well
Nuclei Selected Selected - outer ER Profile 1/2 SER-Edge - Mean per Well
Nuclei Selected Selected - outer ER Profile 2/2 SER-Edge - Mean per Well
Formula Output 2
